# Effectiveness of yoga for major depressive disorder: A systematic review and meta-analysis

**DOI:** 10.3389/fpsyt.2023.1138205

**Published:** 2023-03-23

**Authors:** Yufei Wu, Danni Yan, Jianli Yang

**Affiliations:** Department of Psychology, Tianjin Medical University General Hospital, Tianjin, China

**Keywords:** major depressive disorder, depression, anxiety, yoga, systematic review

## Abstract

**Objective:**

Major depressive disorder (MDD) has a relapse rate that cannot be ignored and places a tremendous burden on the patient in the prevention and treatment process. Yoga, a combination of physical and mental exercises, is effective and acceptable for the adjunctive treatment of MDD. This study aimed to explore further the evidence of yoga’s efficacy for patients with MDD.

**Methods:**

PubMed, Embase, Cochrane library, PsycINFO, SinoMed, CNKI, Wanfang, and VIP databases from their inception to 13 October 2022 were searched by a pre-defined search strategy. RCTs of patients with MDD who met diagnostic criteria for yoga treatment were included. RoB2.0 was used to evaluate the quality of the literature. Improvement in depressive symptoms was assessed by the Beck Depression Inventory (BDI), Hamilton Depression Rating Scale (HAMD), or other scales were used as primary outcome indicators, and improvement in anxiety was assessed by the Hamilton Anxiety Scale (HAMA) and State–Trait Anxiety Inventory (STAI) scale as secondary outcome indicators. RR and Cohen’s d at 95% CI were used as effect size estimates, and Q and I^2^ were used to evaluate the size of heterogeneity, with a *p*-value less than 0.05 indicating statistical significance.

**Results:**

Thirty-four RCT studies, including 1,269 patients in the treatment group and 1,072 patients in the control group, 48.4% of whom were women, were included in the study. Compared to the control group, the BDI-II results yielded a moderate effect of yoga on the improvement of depressive symptoms (Cohen’s *d* = −0.60; 95% CI: −1.00 to −0.21; *p* < 0.01), the HAMD results yielded a moderate improvement of yoga on the severity of depressive symptoms (Cohen’s *d* = −0.64; 95% CI: −0.98 to −0.30; *p* < 0.01), and the STAI results can be concluded that yoga had a negligible effect on the improvement of the level of anxiety (Cohen’s *d* = −0.26; 95% CI: −0.48 to −0.04; *p* = 0.02). No adverse events occurred in the yoga group during the treatment.

**Conclusion:**

Yoga can improve depressive symptoms and anxiety in patients with MDD and has a safe and wide patient acceptance.

**Systematic review registration:**

[https://www.crd.york.ac.uk/prospero/], identifier [PROSPERO, CRD42022373282].

## 1. Introduction

Major depression disorder is a type of depression with a high degree of severity and accompanying symptoms and is one of the most common psychiatric disorders, with a prevalence of 2–20% in the general population (1). According to the surveys of diagnostic data, patients with MDD will experience one or more episodes of depression during their lifetime, with an estimated lifetime prevalence of 16.6% and a relapse rate of between 35 and 80% within 1 year of remission (2, 3). According to statistics, depression ranks as the sixth leading cause of disease in the 10–49 years age group, affecting about 17% of the population, with a considerable disease burden (4). Suicide rates are generally elevated in MDD, especially among younger patients, with at least 10% experiencing suicidal ideation and suicidal behavior (5).

Antidepressant medication (ADM) is currently one of the most commonly prescribed medications for MDD, with 10% or more of the general population taking antidepressants yearly in some high-income countries (6). However, side effects of antidepressants are frequently reported in studies, including central nervous system abnormalities, gastrointestinal reactions, weight changes, and allergic reactions. In one study, electroconvulsive therapy (ECT) was potentially superior to ketamine in improving the severity of depression in the acute phase. Still, both ketamine and ECT have unique adverse reaction profiles (7). In a true sense, relapse prevention is a critical task in the successful treatment of MDD. Clinical guidelines (8) tend to recommend long-term treatment of ADM for relapse prevention and additional psychotherapy for patients with depression at significant risk of relapse, such as those with more previous depressive episodes or those who still have residual symptoms (9). Therefore, continuing psychotherapy or pharmacotherapy after remission or adding psychotherapy sequentially to pharmacotherapy could reduce depression relapse rates during the maintenance of treatment period (10).

Yoga is an integrated model of mind–body practice that includes physical postures, movement, breath control and techniques, relaxation, mindfulness, and meditation (11), which is effective in improving exercise adherence and compliance and is readily accepted by most people (12). In clinical studies, yoga has been applied to Parkinson’s disease (13), chronic pain (14), cancer (15), psychiatric disorders such as anxiety and depression (16, 17), and chronic diseases (18) as an adjunctive therapy. Notable that yoga is well tolerated by patients with MDD while having a high-safety profile (19). However, there is a lack of evidence that yoga is effective in treating MDD. A previous review exploring the effectiveness and safety of yoga interventions for treating patients with depression found some evidence of positive effects superior to placebo (20). Still, the method is unclear due to the small number of randomized trials and patients included in this review. The risk–benefit ratio of the intervention is also dark, meta-analysis cannot be performed, and there are significant limitations. In addition, there was no previous meta-analysis of yoga as an adjunctive treatment for MDD. Consequently, it is necessary to verify the therapeutic effect and safety of yoga on MDD. This study aimed to validate the evidence that yoga could be used as an adjunctive intervention to improve outcomes and reduce relapse rates in the treatment of patients with major depressive disorder.

## 2. Methods

This review was conducted in accordance with the report review under the Preferred Reporting Items for Systematic Evaluation and Meta-Analysis (PRISMA) statement (Supplementary Table 1) (21), and the registration number is CRD42022373282 (22).

### 2.1. Search strategy

The literature search was conducted independently by two researchers, and disagreements were resolved through consultation and discussion with a third researcher. PubMed, Embase, Cochrane library, PsycINFO, SinoMed, CNKI, Wanfang, and VIP databases were searched for randomized controlled trials (RCTs) on yoga for MDD by combining the mesh terms and keywords (“yoga,” “major depression disorder,” etc.). The search time was from each database establishment to 13 October 2022, with no language, region, or sample size restrictions. The PubMed search strategy is shown in Supplementary Table 2.

### 2.2. Eligible criteria

The inclusion criteria are as follows:

1. Type of the study: randomized controlled trials (RCTs) and without language restriction.
2. Type of subjects: (a) All patients with MDD were diagnosed by the Diagnostic and Statistical Manual of Mental Disorders, Fourth Edition (DSM-IV) or the International Classification of Disease 10 (ICD-10). (b) All patients with severe levels were diagnosed by validated clinician-based or self-report depression symptom questionnaire, such as the Hamilton Rating Scale for Depression (23), the Beck Depression Inventory-II (24), or the Center for Epidemiological Studies Depression Scale (CES-D). (c) All patients with major depressive disorder were diagnosed using any other clinician-based diagnosis criterion.

The exclusion criteria are as follows: (a) Type of the study: non-randomized controlled trials (RCTs), duplicate publications, animal studies, reviews, and case reports. (b) Type of subjects: patients diagnosed with other mental disorders.

3. Types of intervention: Yoga intervention measures include breath control suggestions, traditional yoga meditation, or a way of life.
4. Types of outcome measure: (a) Primary outcomes: Improvement in depressive symptom severity was assessed by clinician-rated scales such as the Hamilton Rating Scale for Depression or any other validated scales. Primary indicators included both the BDI-II and the HAMD scale, which assess the degree of depression or symptoms. (b) Secondary indicators: Improvement in anxiety symptoms was assessed by the Hamilton Anxiety Scale (HAMA) or State–Trait Anxiety Inventory (STAI).

### 2.3. Study screening and data extraction

Literature screening was done independently by two authors (Y.F. and D.N.). After abstract title screening, it was then screened by full-text reading, and any disagreements were resolved through discussion. Data extraction included patient information (e.g., age, sex, and diagnosis), method (e.g., randomization), intervention (e.g., type, frequency, and duration of yoga), control intervention (e.g., type, frequency, and time), and outcome (e.g., outcome assessment scales).

### 2.4. Quality assessment

The quality of the literature was evaluated using the Cochrane risk of bias tool (RoB2.0) (25), which includes the bias during randomization, the bias for deviation from established interventions, the bias for missing outcome data, the bias for outcome measures, the bias for selective reporting of outcomes, and an overall bias. The evaluation levels include low risk, high risk, and some concerns. Publication bias refers to the fact that among similar studies, the results of studies with negative statistical significance are more difficult to be published than those with positive statistical significance. Visual analysis of funnel plots was used to assess the symmetry of this study, and Egger testing was used to quantify publication bias. The study sample size increases the accuracy of estimates of intervention effectiveness. Therefore, the estimated value of efficacy of the study with a large sample size is narrow at the bottom of the funnel plot, while the study with a small sample size is more scattered. Egger’s and Begg’s test and funnel plots are shown in Supplementary Figure 2 and Supplementary Table 4.

### 2.5. Risk of bias

**Figure 1:**
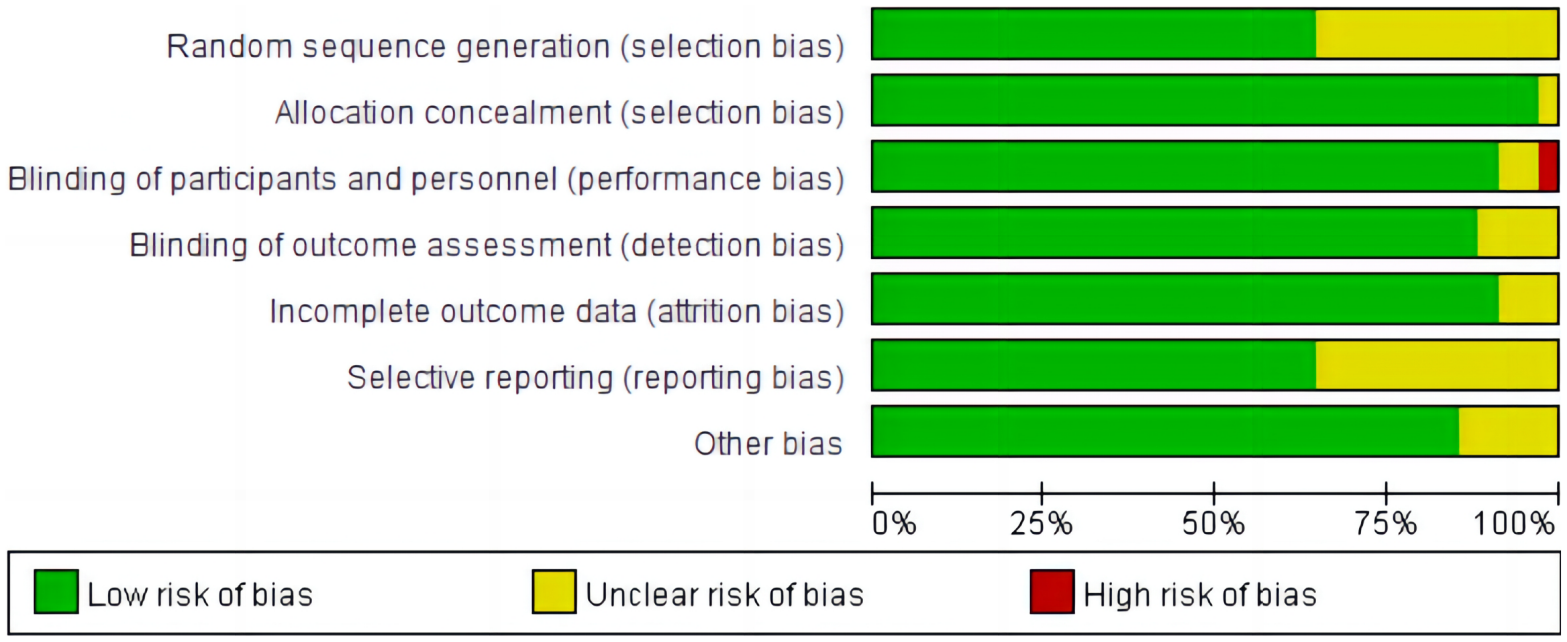
Risk of bias graph: review authors’ judgments about each risk of bias item, presented as a percentage of included studies.

**Figure 2:**
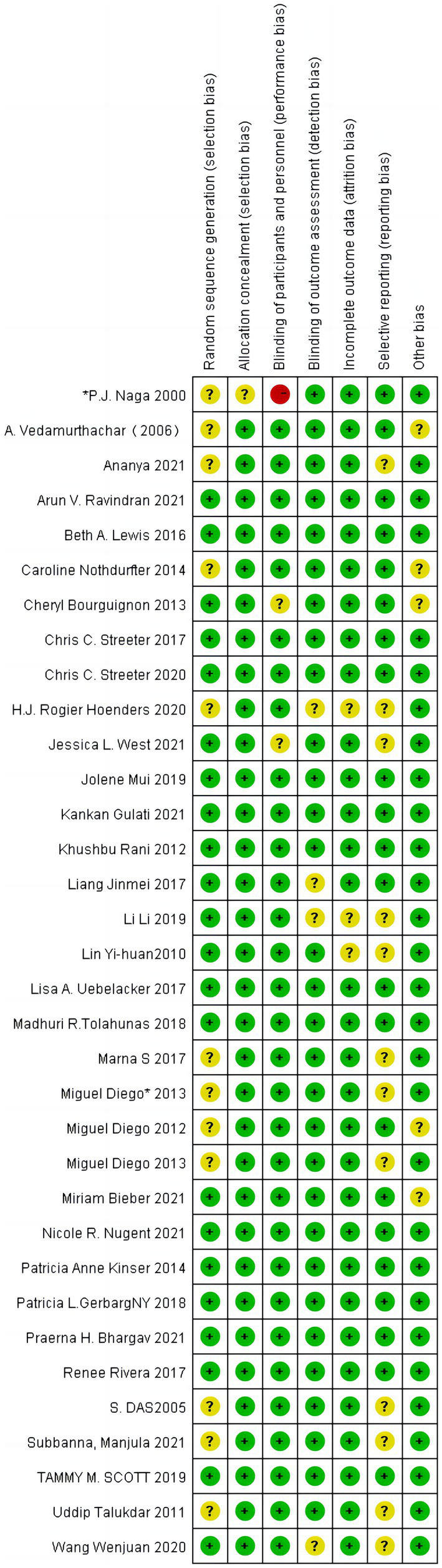
Risk of bias summary: a review of the authors’ judgments about each risk of bias item for each included study.

All studies were assessed as biased with uncertain risk for at least one domain. Figures 1, 2 show an overall risk of bias assessment across domains and the risk of bias in each included study. There was not enough information in 12 studies to judge whether the studies were at high risk or low risk for selection bias. Researchers rated five studies as uncertain risks for bias owing to participant exclusion without explanation, loss of follow-up imbalance, self-selection bias, self-reported compliance, and a lack of clarity in the management of yoga interventions to measure outcomes.

### 2.6. Assessment of the quality of the evidence—GRADE

Grading of Recommendations Assessment, Development and Evaluation (GRADE) was used to evaluate the quality of data evidence. Evidence from low- and moderate-quality studies suggests yoga interventions are effective for each of the outcomes examined. A detailed assessment of the evidence is contained in Supplementary Figures 3A–F.

### 2.7. Data analysis

After extracting the data, two researchers decided which outcome to include in the meta-analysis that was performed by Stata 17.0 software. We calculated and compared the standardized mean difference (Cohen’s d), standard deviation (SD), or 95% confidence interval (95% CI), and the number of participants for each intervention group before and after the intervention (pre and post) in each study. Heterogeneity was evaluated using the I^2^ and Q test. *I*^2^ < 25% was considered low heterogeneity, between 25 and 50% was considered moderate heterogeneity, and > 75% was considered significant heterogeneity. It is generally believed that *I*^2^ > 50% indicates apparent heterogeneity, and the random effects model is used. If *I*^2^ ≤ 50%, the fixed effects model is used (26). In this study, we used random effects models. The research results were represented in a forest plot. In addition, meta-regression or subgroup analysis was conducted to evaluate meta-analysis with high heterogeneity (*I*^2^ > 75%).

### 2.8. Sensitivity analysis

A sensitivity analysis shall be conducted with the results to determine the robustness of the results. We conducted a sensitivity analysis on the results one by one and generate sensitivity figures, representing the result of removing the total effect from each article. Excluded items may result in biased results, i.e., deleted things can significantly impact the actual outcome and re-merge the data. The meta-analysis results are relatively stable if there is no substantial change in the pre- and post-combined effect. If there are significant differences or even diametrically opposite conclusions then in that case, the stability of the results of meta-analysis is worse, and it is necessary to be careful when interpreting the results and making a conclusion.

### 2.9. Subgroup analysis

We conducted four subgroup analyses of studies with the Hamilton Depression Assessment Scale as the primary outcome to investigate the impact of specific geographic location, type, duration of intervention, and frequency of intervention on the effectiveness of improving depressive symptoms or degrees in patients with major depressive disorder.

## 3. Results

### 3.1. Literature search

**Figure 3:**
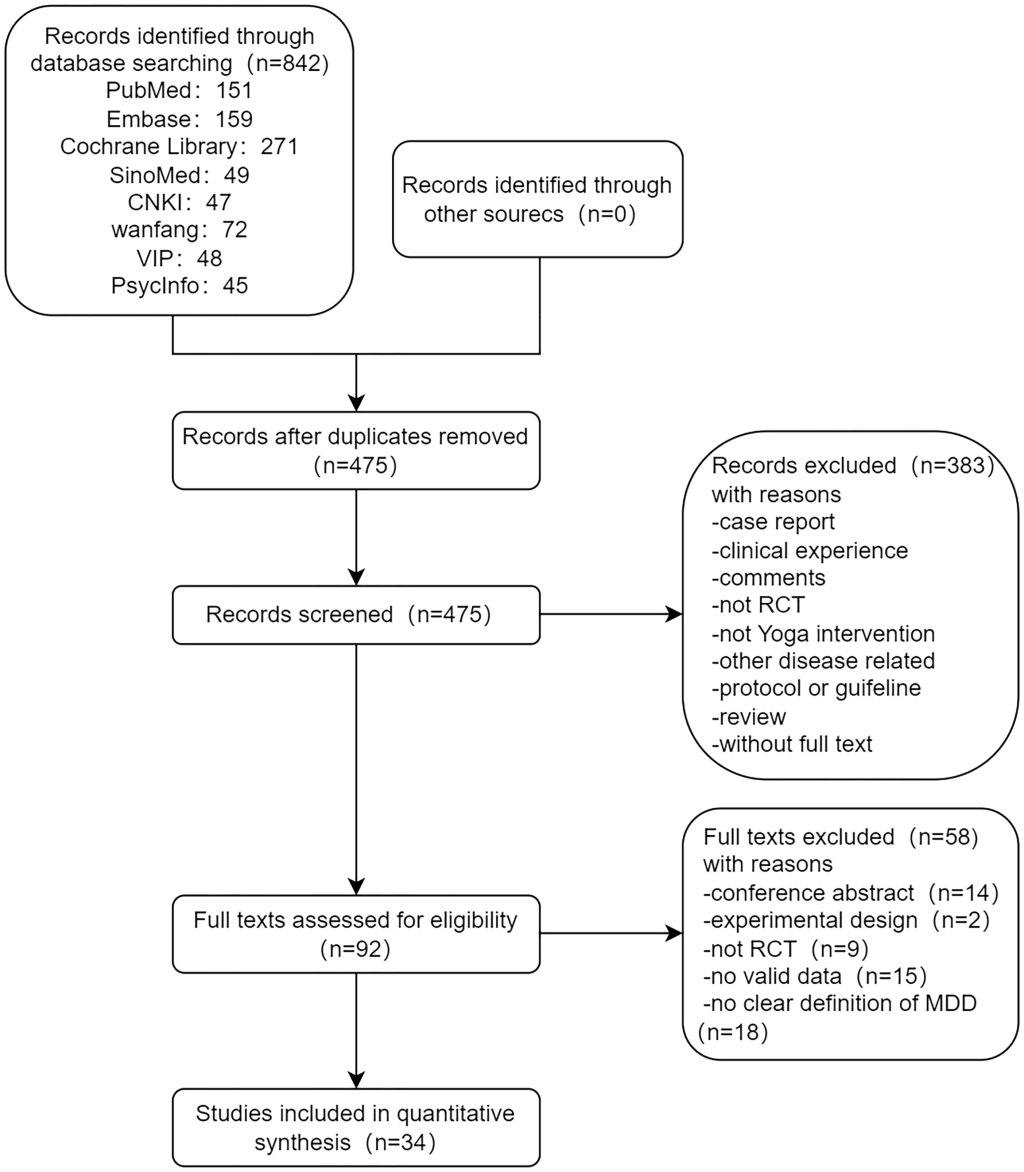
Literature screening flowchart.

The literature search initially detected 842 studies, and after stepwise screening based on the title, abstract, and full text, 34 studies were finally included (19, 27–59). This included 1,269 patients in the treatment group and 1,072 patients in the control group, which met our pre-defined inclusion criteria and were included in the final meta-analysis. The literature screening flowchart is shown in Figure 3.

### 3.2. Characteristics

Of the 34 RCTs included, 11 (27–30, 32, 43, 44, 47, 49, 52, 53) were from India, 13 (31, 33, 35, 36, 38–42, 46, 50, 54, 55) were from the United States, two (37, 38) were from Germany, two (19, 34) were from the United Kingdom, one (51) was from Canada, and five (45, 56–59) were from China. One study (29) included only male participants, nine studies (31, 33–36, 38, 55, 57, 59) included only female participants, three studies (31, 33, 34) had only women with prenatal depression, and one study (59) included postpartum depression. Eleven RCTs (19, 27, 28, 37, 40, 42, 49, 50, 53, 55) included patients with a DSM-IV diagnosis of MDD, six RCTs (41, 48, 52) included patients with a score greater than or equal to 24 on the Hamilton Depression Inventory for patients with MDD, two RCTs (33, 44) included patients with DSM-V diagnosis of MDD, and two RCTs (35, 36) included patients with the MINI diagnosis of MDD. Of the four studies from China, two (56, 57) met the CCMD-3 diagnostic criteria for depression, one (58) used the CCMD-10 diagnostic criteria, and the others (59) included the EPDS diagnostic criteria for depression. The mean age of the patients in the intervention group was 34.66 years, the mean age of the patients in the control group was 31.13 years, and the median age was 32.90 years. Female patients in each study ranged from 0 to 100.0% (median: 54.12%) (Table 1).

**Table 1:** Characteristics of the included studies.

Study	Patients (N, diagnosis, age)	Co-intervention (drug or psychotherapy)	Intervention		Outcomes	Result
			Treatment	Control		
P.J. Naga Venkatesha Murthy, India (2000)	45 subjects with depression based on DSM-IV criteria, scored 17 or more on the total HSRD-17. Yoga: 36.0 (7.8) years Males/Females: 9/6 ECT: 36.7 (2.5) years Males/Females: 6/9 IMN: 43.4 (11.9) years Males/Females:10/5	No	Sudarshan Kriya Yoga Imipramine (IMN)	Electroconvulsive therapy (ECT)	1. BDI 2. HRSD (wk 0, 4)	Significant reductions in the total scores on Beck Depression Inventory (BDI) and Hamilton Rating Scale for Depression (HRSD) occurred on successive occasions in all three groups.
S. DAS, S. MONDAL, New Delhi (2005)	30 patients suffering from Major depression with DSM-IV criteria. Yoga: 31.87 ± 8.78 years Control: 31.67 ± 8.46 years Males/Females: 19/11	Yes	Sahaj Yoga plus Conventional antidepressant medication	Conventional antidepressant treatment plus simple position and eyes closed	1. HAM-D 2. Neuro-cognitive test battery consisting of Letter cancelation test (LCT) 3. HAMA	Table II demonstrates percentage reduction in HAM-D scores at 8 weeks was significantly more in Group 1 patients than in Group 2 patients (*p* = 0.003).
A. Vedamurthachar, Bangalore, India (2006)	60 patients All males mean age: 39.7 ± 5.8 years	Not mention	Sudarshana Kriya Yoga (SKY)	Not	1. BDI	Both groups reductions in BDI scores occurred but significantly more so in the SKY group.
Uddip Talukdar, India (2011)	30 patients, mean age 35 years M/F: 9/21	Not mention	Himalayan Yoga counseling	General	1. BDI 2. BAI 3. CGI-S, CGII, GAF	Changes were more significant in pre- and post-assessment of the experimental group.
Miguel Diego, USA (2012)	84 patients all females mean age: 26.6 years	Not mention	Yoga therapy	Massage	1. CES-D 2. STAI 3. STAIX	A greater decrease on depression, anxiety, and back and leg pain scales and a greater increase on a relationship scale.
Khushbu Rani, SC Tiwari, India (2012)	126 patients Yoga (65): 27.67 ± 7.85 years, Control (61): 26.58 ± 6.87 years	Yes	Yoga Nidra therapy Plus pharmacotherapy	Only pharmacotherapy	1. HAMD 2. HAMA	No significant improvement in the patients with severe anxiety and depressive symptoms.
Miguel Diego, PhD, USA (2013)	92 pregnant women patients diagnosed with SCID. mean age: 24.9 ± 5.2 years	Not mention	Yoga support	Social support	1. CES-D 2. STAI 3. STAIX 4. Relationship	They both had lower depression (CES-D), anxiety (STAI), and anger (STAXI) scores and improved relationship scores.
Miguel Diego, US (2013)	92 pregnant women patients diagnosed with SCID. Mean age: 26.6 ± 5.5 years	Not mention	Yoga/Tai chi	Waitlist control	1. SCID 2. CES-D 3. STAI	The tai chi/yoga group had lower summary depression (CES-D) scores, as well as lower negative affect and somatic/vegetative symptoms subscale scores on the CES-D, lower anxiety (STAI) scores and lower sleep disturbances scores
Cheryl Bourguignon, PhD, Charlottesville, VA, USA (2013)	27 MDD women with a current major depressive episode or dysthymia using the MINI-International Neuropsychiatric Interview (MINI) Yoga (15): 40.93 ± 15.84 years Control (12): 46.17 ± 15.40 years	Not mention	Yoga	Attention-control group	1. PHQ-9 2. STAI 3. PSS 4. RRS	There was a decrease in depression over time in both the yoga group and the attention control group
Patricia Anne Kinser, PhD, Richmond, USA (2014)	27 MDD women diagnosed with MINI. Yoga: 40.9 ± 15.8 years Control: 46.2 ± 15.40 years	Not mention	Hatha yoga	Health-education (HE)	1. PHQ-9| 2. PSS-10 3. STAI 4. RRS 5. SF-12	Whether or not an individual continues with yoga practice, simple exposure to a yoga intervention appears to provide sustained benefits to the individual
Caroline Nothdurfter, Germany (2014)	60 inpatients suffering from MDD according to DSM-IV M/F: 38/15	Yes	QXR (300 mg/day) therapy or ESC (10 mg/day)	Hatha yoga	1. HAMD	Antidepressant agents down regulate HPA axis function to a greater extent than additional Hatha yoga treatment.
Beth A. Lewis, USA (2016)	40 women who met the criteria for depression based on (SCID-I), Yoga: 45.55 (12.30) years Control: 39.8 (11.23) years	Not mention	Yoga	Walking control condition	1. BDI 2. RRS	Both groups reported decreases in depressive symptoms from baseline to post-intervention.
Renee Rivera, San Francisco (2017)	38 patients with MDD, 68% Female mean age: 43.4 ± 14.8 years	Not mention	Hatha yoga	Attention control education groups	1. BDI 2. GSES/RSES	Yoga participants exhibited significantly greater 8 week decline in BDI scores than control groups.
Marna S. Barrett, PhD, Philadelphia, USA (2017)	25 Patients with MDD met the criteria on DSM-IV-TR Yoga (13): 39.4 (13.9) years Control (12): 34.8 (13.6) years M/F: 7/18	Not mention	Sudarshana Kriya yoga	Waitlist controlled	1. HDRS-17 2. BDI 3. BAI	The SKY arm (*n* = 13) showed a greater improvement in HDRS-17 total score compared to waitlist control
Chris C. Streeter, Boston, USA (2017)	30 patients with MDD HRSD scores>17 HDG: Mage: 38.4 ± 15.1 years LDG: Mage: 34.7 ± 10.4 years M/F: 5/25	Not mention	High dose group	Low dose group	1. BDI-II	Depressive symptoms declined significantly in patients with MDD in both the HDG and LDG. Both groups showed comparable compliance and clinical improvements, with more subjects in the HDG exhibiting BDI-II scores 10 at week 12.
Lisa A. Uebelacker, Ph.D. Providence, RI, USA (2017)	122 subjects met criteria for major depressive disorder (MDD) within the prior 2 years assessed *via* the Structured Clinical Interview for DSM-IV. Yoga: mean age 46.78 HLW: mean age 46.2	Not mention	Yoga group	Healthy living workshop	1. QIDS 2. PHQ-9	1. Not find a statistically significant difference between groups in depression symptoms. 2. Fifty-one percent of Yoga participants demonstrated a response (50% reduction in depression symptoms)
Patricia L. Gerbarg, NY, US (2018)	32 Patients diagnosis of MDD using DSM-IV criteria LDG: 34.7 ± 10.4 years HDG: 38.4 ± 15.1 years M/F: 5/25	Not mention	High dose group	Low dose group	1. BDI-II 2. The C-SSRS item 1	At screening, SI without intent was endorsed on the BDI-II by 9 participants; after completing the intervention, 8 out of 9 reported resolution of SI.
Madhuri R. Tolahunas, India (2018)	178 Patients with MDD according to DSM-V Yoga (89): 38 ± 9 years Drug (89): 40 ± 8 years M/F: 93/85	No	Yoga-based intervention	Drug group lifestyle	1. BDI-II	YBLI provides MDD remission in those who have susceptible 5-HTTLPR and MTHFR 677C > T polymorphisms and are resistant to SSRIs treatment. YBLI may be therapeutic for MDD independent of heterogeneity in its etiopathogenesis.
Madhuri R. Tolahunas, India (2018)	58 MDD patients diagnosed with DSM-V criteria. Yoga: 36.94 (8.94) years Control: 39.10 (9.26) years M/F: 27/31	No	YMLI program plus routine drug treatment	Only routine drug treatment	1. BDI-II	These results suggest that a decrease in depression severity after YMLI in MDD is associated with improved systemic biomarkers of neuroplasticity.
Jolene Mui, Hongkong (2019)	32 participants diagnosed with current MDD (scores between 14 and 28 on BDI) HDG (15): 38.4 ± 15.1 years LDG (15): 34.7 ± 10.4 years M/F: 5/25	No	Laughter group	Treatment as usual	1. The Depression Anxiety Stress Scale (DASS–21) 2. CSQ-8	The LY group had statistically greater decreases in depression and improvements in mental health related quality of life compared to the control group from T0 to T1. The CSQ-8 scores indicated a favorable level of satisfaction with the LY intervention.
TAMMY M. SCOTT, PhD, Boston (2019)	Patients diagnosed with current MDD BDI-II scores between 14 (mild depression) and 28 (severe depression) LDG (15): 34.67 ± 10.38 years HDG (15): 39.62 ± 15.61 years Female (%) 80.0/84.6 (LDG/HDG)	Not mention	High dose group	Low dose group	1. Positivity Self-Test (PST) 2. STAI 3. Patient Health Questionnaire (PHQ-9)	Significant improvements in all outcome measures were found for both groups, with acute and cumulative benefits.
Chris C. Streeter, MD, Boston (2020)	171 females met a primary diagnosis of MDD based on DSM-IV mean age 25.08 (4.64) years	Not mention	High dose group	Low dose group	1. BDI-II 2. Thalamic GABA levels	BDI-II scores improved significantly in both groups. GABA levels from Scan-1 to Scan-3 and from Scan2 to Scan-3 were significantly increased in the LDG (*n* = 15) and showed a trend in the total cohort.
Praerna H. Bhargav, India (2021)	83 patients YG: mean age 48.38 (10.21) years TAU: mean age 51.31 (9.19) years M/F: 66/17	Not mention	Yoga group	Waitlist group	1.HDRS-17	Clinically both groups improved significantly over time with more improvement and higher remission rate in YG (62.8%) than WG (45.7%).
Miriam Bieber, Germany (2021)	68 subjects scoring ≥18 on 17-item Hamilton Depression Rating Scale (HDRS) mean age: 31.58 ± 8.79 years M/F: 28/40	Yes	Ashtanga-Yoga	Waiting-list control group: TAU	1. BDI-II MADRS 2. Positive and Negative Affect Scale (PANAS)	Remission rates indicated a significant improvement in the yoga group (BDI-II: 46.81%, MADRS: 17.02%) compared to the control group (BDI: 33.33%, MADRS: 8.33%).
Kankan Gulati NIMHANS, India (2021)	87 patients met criteria for MDD *via* DSM-IV SCID mean age: 45.20 (12.72) years female (84%)	Not mention	Yoga	Waitlist group	1. HDRS-17 2. HRV variable	Findings suggest Yoga therapy may help in bringing parasympathetic dominance in patients with MDD.
Nicole R. Nugent, Rhode Island Hospital, USA (2021)	72 patients met DSM-IV criteria for major depression Receiving Yoga First: 39.36 (11.69) years Receiving Psychoeducation First: 40.58 (12.72) years M/F: 15/57	Not mention	Hatha yoga	Health-education (HE) control group	1. QIDS 2. IL-6 and TNF-α levels	We observed a significant reduction in IL-6 concentrations in the yoga treatment group relative to the health education control group, as demonstrated by a negative interaction between treatment group and slope of IL-6.
Arun V. Ravindran, Toronto (2021)	81 patients diagnosed MDD based on ICD-10 Intervention (29): 30.48 (10.22) years Control (52): 33.61 (8.97) years M/F: 39/42	Yes	Yoga (8 weeks) Psychoeducation (8-16 weeks)	Psychoeducation (8 weeks) follow. Yoga (8–16 weeks)	1. MADRS (primary) 2. HAMD, CGI, BDI, QLESQ, PSS (Secondary)	1. There was a significant decline in depressive symptoms, as measured by the MADRS, following 8 weeks of yoga. 2. There was no significant difference in MADRS ratings between intervention groups.
Ananya Srivastava, India (2021)	22 subjects scoring ≥18 on HAMD without details about mean ages of subjects	Yes	Psychotropic medications plus Kriya yoga	Only psychotropic medications	1. HDRS-17	HDRS scores of the intervention group (*n* = 29) were found to be significantly lesser than that of the control group (*n* = 52) by the end of 2, 4, and 8 weeks.
Subbanna, Manjula, India (2021)	110 subjects met criteria for MDD by DSM-IV mean age of 47.2 (11.8) years female (86%)	Yes	Yoga therapy (YT)	Waitlist control	1. HAMD 2. MADRS 3. CGI	Yoga therapy down regulating the plasma levels of C1q, Factor H, and properdin seem quite interesting.
Jessica L. West, Duke University Medical Center (2021)	32 participants diagnosed with current MDD, scores on BDI-II between 14 and 28 HDG (15): 38.4 ± 15.1 years LDG (15): 34.7 ± 10.4 years M/F: 5/25	Not mention	Yoga	HLW classes	1. QIDS 2. FFMQ 3. RSQ	A small effect of yoga on components of mindfulness during a 10 week intervention period.
Lin Yi-huan, Wang Jun-qing, China (2010)	60 subjects scoring ≥24 on HAMD, Intervention (30):26.353 (4.62) years, M/F:9/21;Controls (30):28.08 (6.23) years, M/F:11/19	Yes	Yoga and psychotropic medications	Psychotropic medications	1. HAMD	There was significant difference in HAMD scores between intervention groups and control groups
Wang Wenjuan, Pan Qin, Zhang Huimin, China (2020)	41 subjects scoring ≥35 on HAMD, All Females	Not mention	Yoga	Treatment as usual	1. HAMD GSES	There was significant difference in HAMD and GSES scores between intervention groups and control groups
Liang Jinmei, Kang Xingxing, Ji Wei, China (2017)	92 subjects scoring ≥24 on HAMD, Intervention (44):37.1 (4.6) years, M/F:19/25;Controls (44):29.0 (4.9) years, M/F:20/24	Yes	Yoga and psychotropic medications as usual	Psychotropic medications as usual	1. HAMD	There was significant difference in HAMD scores between intervention groups and control groups
Li Li, Gao Lin, China (2019)	100 subjects scoring ≥17 on EPDS, intervention (50):26.71 (5.38) years, controls (50):26.21 (6.31) years, all females	Not mention	Yoga and psychotherapy	Routine perinatal care and psychotherapy	1. EPDS SAS	There was significant difference in EPDS and SAS scores between intervention groups and control groups

Among all randomized controlled trials included, four (19, 41, 46, 55) used Iyengar yoga, seven (35–37, 39, 42, 43, 50) used Hatha yoga, eight (30, 38, 44, 51, 56–59) used self-designed yoga based on breathing, meditation, and mindfulness, four (27, 29, 40, 52) used Sudarshan Kriya yoga, three (31, 33, 34) used yoga specially designed for the middle and late pregnancy, one (28) used SAHAJ yoga, one (48) used Ashtanga yoga, one (45) used Laugher yoga, one (32) used Yoga Nidra, and four (47, 49, 53, 54) studies did not mention yoga methods. Seven studies (28, 32, 37, 44, 52, 56, 58) added yoga as an adjunct to regular treatment, nine studies (28, 35, 36, 39, 40, 52, 56–58) had a duration of 8 weeks, 14 studies (19, 31, 33, 34, 38, 41, 43, 44, 46–49, 53, 55) had a more extended study duration of 12 weeks, and one study (41) had a maximum period of 28 months. The mean study duration of the studies included here was 10 weeks. Combined pharmacological/psychotherapeutic interventions were allowed in 11 studies (28, 32, 37, 48, 51–53, 56, 58) and no combined intervention in four RCTs (27, 43–45). The specific characteristics of the included studies are detailed in Table 2.

**Table 2:** Types of the invention used in included studies in the meta-analysis.

Study	Minutes	Frequency	Type	Duration	Control
P.J. Naga Venkatesha Murthy, India (2000)	45 min	4 × wk	Sudarshan Kriya Yoga	4 weeks	Electroconvulsive therapy (ECT): bilateral electrode, 3 × wk. Imipramine (IMN): 150 mg/daily
S. DAS, New Delhi (2005)	30 min	3 × wk	Sahaj yoga	8 weeks	Conventional antidepressant treatment +simple position and eyes closed
A. Vedamurthachar, Bangalore, India (2006)	60 min	Not mention	Sudarshana Kriya Yoga	2 weeks	Not controls
Uddip Talukdar, India (2011)	60 min	4 weeks; 5 ~ 28 wk	Yoga	28 weeks	General counseling: 30 min, 1 × wk
Miguel Diego, USA (2012)	20 min	2 × wk	Yoga	12 weeks	Massage: 20 min, 2 × wk
Khushbu Rani, SC Tiwari, India (2012)	35 min	5 × wk	Yoga Nidra	six months (24 weeks)	Only pharmacotherapy
Miguel Diego, PhD, USA (2013)	20 min	1 × wk	Yoga	12 weeks	Social support: control for the possible effects of attention and social support gained by women 1 × wk.
Miguel Diego, US (2013)	20 min	1 × wk	Tai chi/Yoga	12 weeks	Waitlist control group: 20 min; 1 × wk
Cheryl Bourguignon, PhD, RN	75 min	1 × wk	Yoga	8 weeks	Attention-control group: 75 min; 1 × wk
Patricia Anne Kinser, PhD, Richmond (2014)	75 min	1 × wk	Yoga	8 weeks	Health-education (HE) control group
Caroline Nothdurfter, Germany (2016)	60 min	1 × wk	QXR (300 mg/day) or ESC (10 mg/day)	5 weeks	Hatha yoga
Beth A. Lewis, USA (2016)	60–75 min	2 × wk	Yoga	12 weeks	Walking control condition: 2 × wk
Renee Rivera, San Francisco (2017)	90 min	2 × wk	Hatha yoga	8 weeks	Attention control education groups: 90-min; twice weekly for 8 weeks.
Marna S. Barrett, PhD, Philadelphia (2017)	3.5 h-week 1 1.5 h + 20 ~ 25 min/d: week 2 ~ 8	Week 1: 6 × wk., 3.5 h/d week 2–8:1 × wk. + home practice version of SKY (20–25 min per day)	Sudarshan Kriya yoga	8 weeks	Waitlist control group
Chris C. Streeter, Boston (2017)	Three 90-min yoga classes +four 30-min homework sessions	1 × wk	HDG	12 weeks	LDG: two 90-min yoga classes and three 30-min homework sessions/wk
Lisa A. Uebelacker, Ph.D. Providence, RI (2017)	80 min	2 × wk	Yoga	10 weeks	HLW classes: 1–2 × wk. 60 min/session
Patricia L. Gerbarg, NY (2018)	90 min	1 × wk	High dose group	12 weeks	The LDG included two 90-min yoga classes plus three 30-min homework sessions weekly.
Madhuri R. Tolahunas, India (2018)	120 min	5 × wk	YBLI program	12 weeks.	Drug: Escitalopram (5–20 mg/day) fluoxetine (20–40 mg/day), paroxetine (20–40 mg/day) were used in 36, 24, and 29, patients, Adjunctive medications were prescribed for sleep (diphenhydramine, 25–50 mg; or zolpidem, 5–10 mg), and for anxiety (clonazepam, 0.25 mg twice daily; or lorazepam, 0.5 mg 3 times/day)
Madhuri R. Tolahunas, India (2018) 58 MDD	120 min	1 × wk	YBLI program	12 weeks	Control: routine drug treatment
Jolene Mui, Hongkong (2019)	45 min	2 × wk	Laughter Yoga (LY)	4-weeks	Control: TAU (Treat as usual): received their usual routine community mental health care (including medications) and attended medical outpatient appointments as determined by their individual needs.
TAMMY M. SCOTT, PhD, Boston (2019)	Three 90-min classes plus four 30-min homework sessions	1 × wk	HDG	12 weeks	LDG: two 90-min yoga classes and three 30-min homework sessions/wk
Chris C. Streeter, MD, Boston (2020)	Three 90-min classes plus four 30-min homework sessions	1 × wk	HDG	12 weeks	LDG: two 90-min yoga classes and three 30-min homework sessions/wk
Praerna H. Bhargav, India (2021)	60 min	12 wk	Yoga	12 weeks	WG was offered yoga after the trial period of 12 weeks.
Miriam Bieber, Germany (2021)	90 min	3 × wk	Ashtanga-Yoga	12 weeks	TAU: consisting of antidepressant medication and psychotherapy.
Kankan Gulati NIMHANS, India (2021)	60 min	12 sessions in the first 4 weeks two booster sessions in next 8 weeks+monitored home based practice - 4 days/week.	Yoga	12 weeks	WG: to learn yoga after 12 weeks.
Nicole R. Nugent, Rhode Island Hospital (2021)	80 min	2 × wk	Hatha yoga	10 weeks	Control: health education: 2/wk., 60 min, 10 weeks
Arun V. Ravindran, Toronto (2021)	90 min	2 × wk	8 weeks of yoga and 8 weeks of psychoeducation	16 weeks	8 weeks of psychoeducation, followed by yoga for equal duration
Ananya Srivastava, India (2021)	45 min	Daily × first 2 weeks home practice 20 min	Kriya Yoga	8 weeks	Control: only psychotropic medications
Subbanna, Manjula, India (2021)	Not	4–6/wk	Yoga	12 weeks	Given antidepressants as per the routine clinical treatment guidelines.
Jessica L. West, Duke University Medical Center (2021)	80 min	1 × wk	Yoga	10 weeks	HLW classes: 60 min, 1 × wk
Lin Yi-huan, Wang Jun-qing, China (2010)	60 min	4 × wk	Yoga	8 weeks	Control: psychotropic medications as usual
Wang Wenjuan, Pan Qin, Zhang Huimin, China (2020)	90 min	4 × wk	Yoga	8 weeks	Control: treatment as usual
Liang Jinmei, Kang Xingxing, Ji Wei, China (2017)	45 min	5 × wk	Yoga	8 weeks	Control: psychotropic medications as usual
Li Li, Gao Lin, China (2019)	60 min	2 × wk	Yoga	8 weeks	Control: treatment as usual and psychotherapy

### 3.3. Methodological quality

Of the 34 included RCTs, 22 studies mentioned randomization (19, 32, 35, 36, 38, 39, 41–47, 49–51, 54–59), 13 studies reported allocation concealment (19, 32, 39, 41, 43, 46, 47, 49, 55–59), 18 studies mentioned blinding (three single-blind (32, 41, 51), four studies mentioned double-blind (39, 42, 50, 54), 11 studies mentioned unblind (27, 38, 40, 43–47, 52, 55)), and the remaining six studies did not mention blinding; all 35 studies clearly had no other sources of bias and selective reporting of risk (Supplementary Table 3).

### 3.4. Meta-analysis

**Figure 4:**
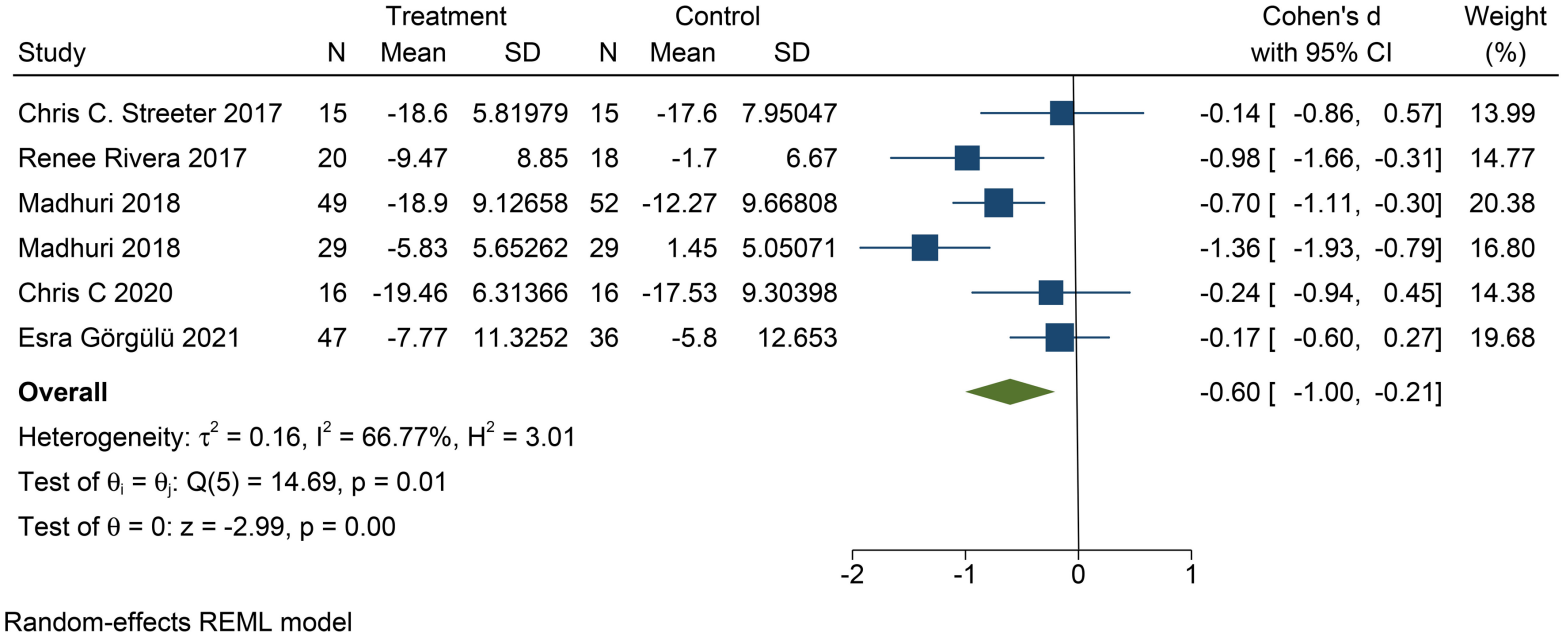
Forest plot of BDI-II.

**Figure 5:**
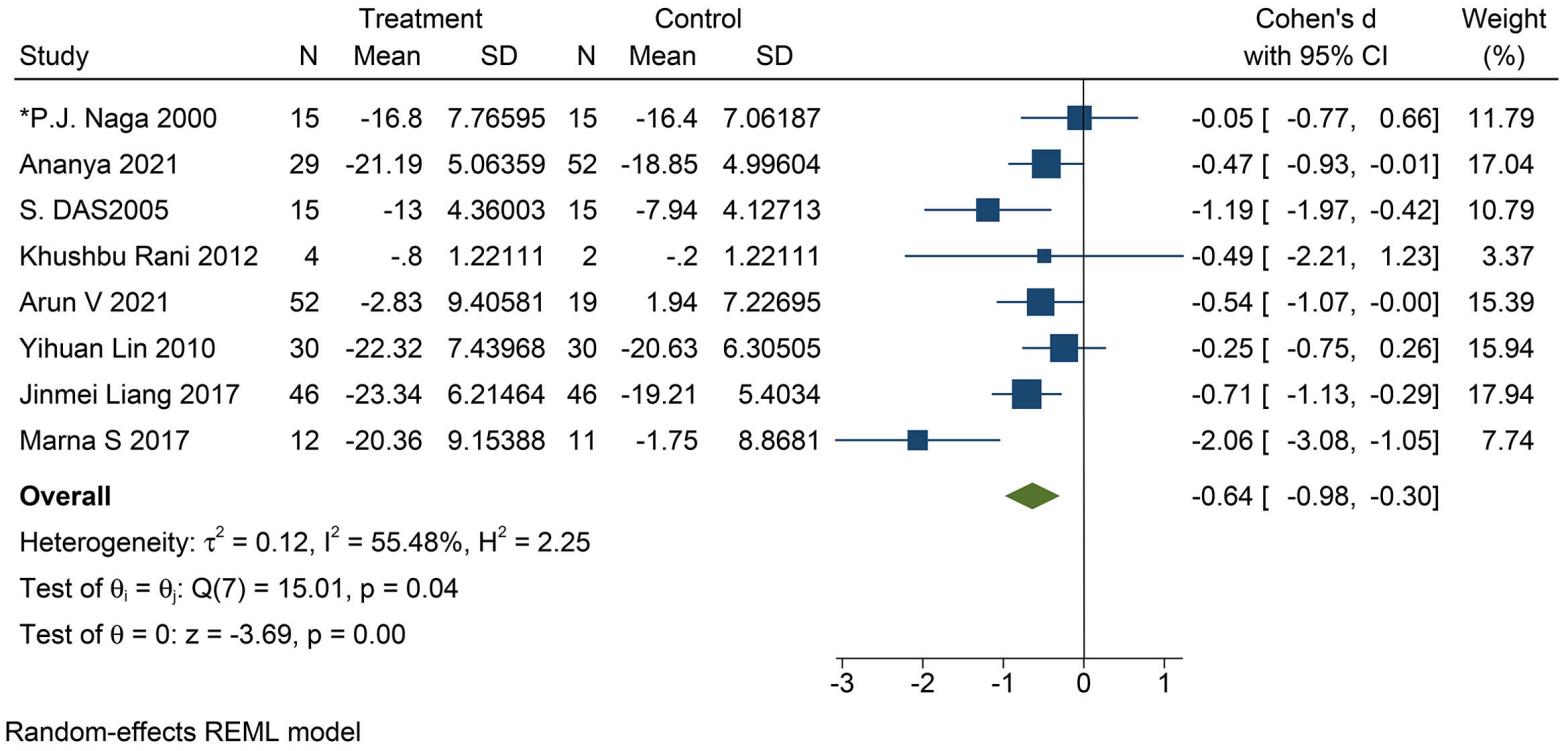
Forest plot of HAMD.

**Figure 6:**
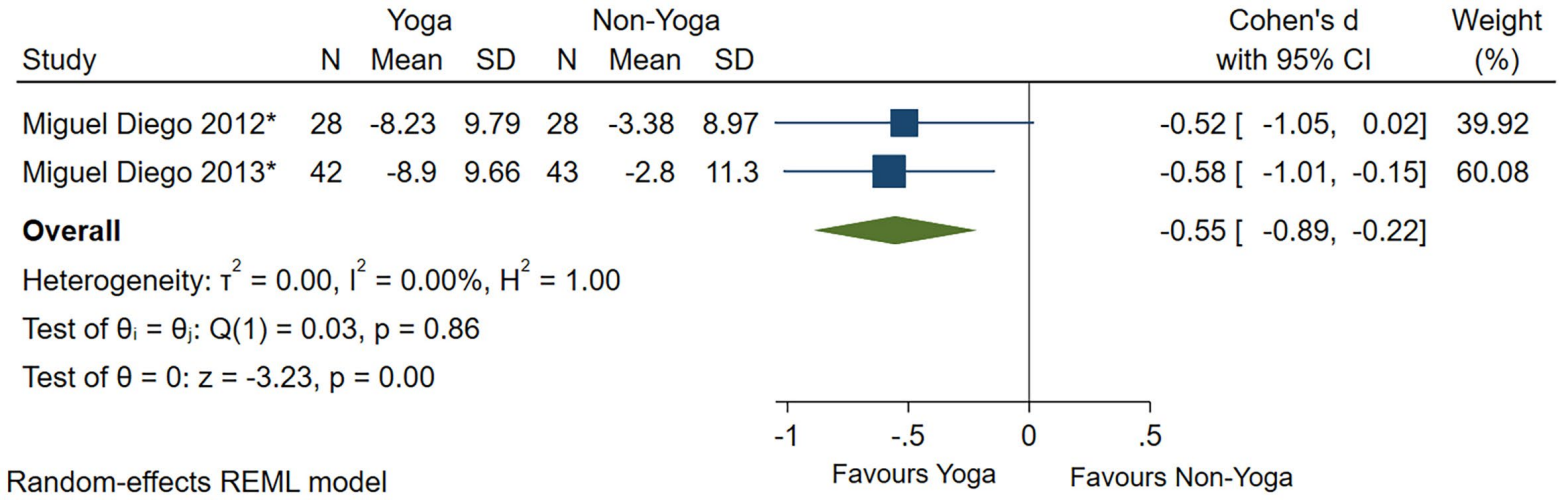
Forest plot of CES-D.

The improvement in depressive symptom severity was assessed separately by clinician-administered rating scales, and six studies yielded moderate effects of yoga on depressive symptom severity when assessed by the BDI-II (Cohen’s *d* = −0.60; 95% CI: −1.00 to −0.21; *p* < 0.01; heterogeneity: *I*^2^ = 66.77%; *Q* (5) = 14.69, *p* = 0.01) (Figure 4). Eight studies could conclude moderate improvement of yoga on the severity of depressive symptoms when assessed by HAMD (Cohen’s *d* = −0.64; 95% CI: −0.98 to −0.30; *p* < 0.01; heterogeneity: *I*^2^ = 55.48%; *Q* (7) = 15.01, *p* = 0.04) (Figure 5). When the two studies were assessed by CES-D, it could be concluded that yoga had a moderate improvement in the severity of depressive symptoms (Cohen’s *d* = −0.55; 95% CI: −0.89 to −0.22; *p* < 0.01; heterogeneity: *I*^2^ = 0.00%; *Q* (1) = 0.033, *p* = 0.86) (Figure 6).

**Figure 7:**
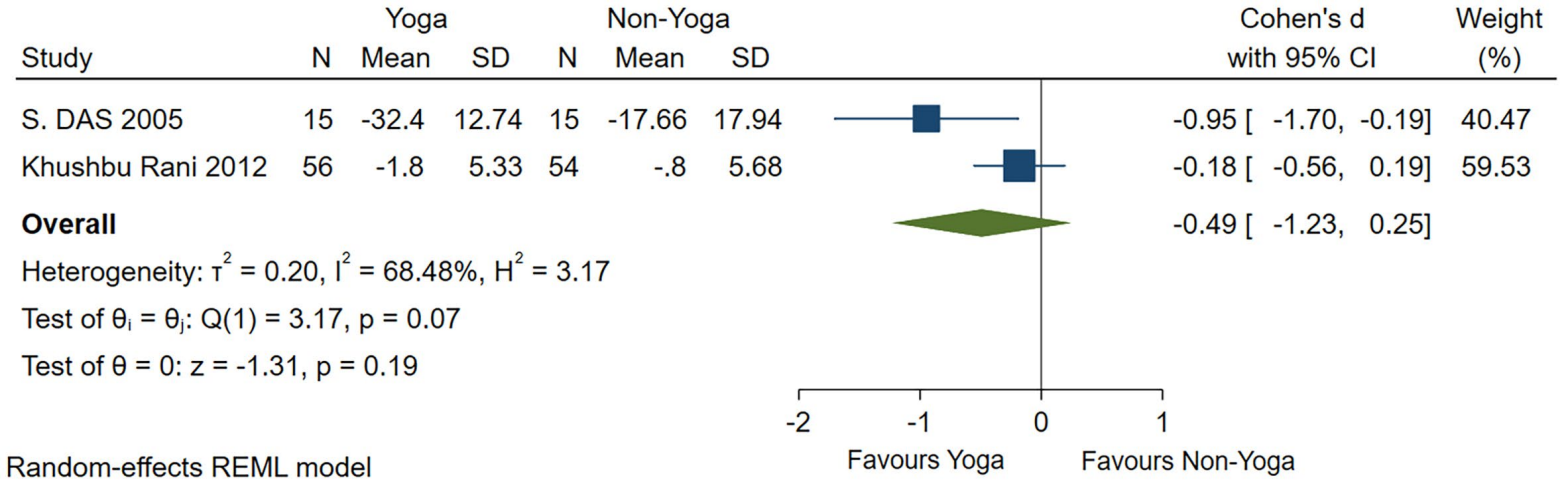
Forest plot of HAMA.

**Figure 8:**
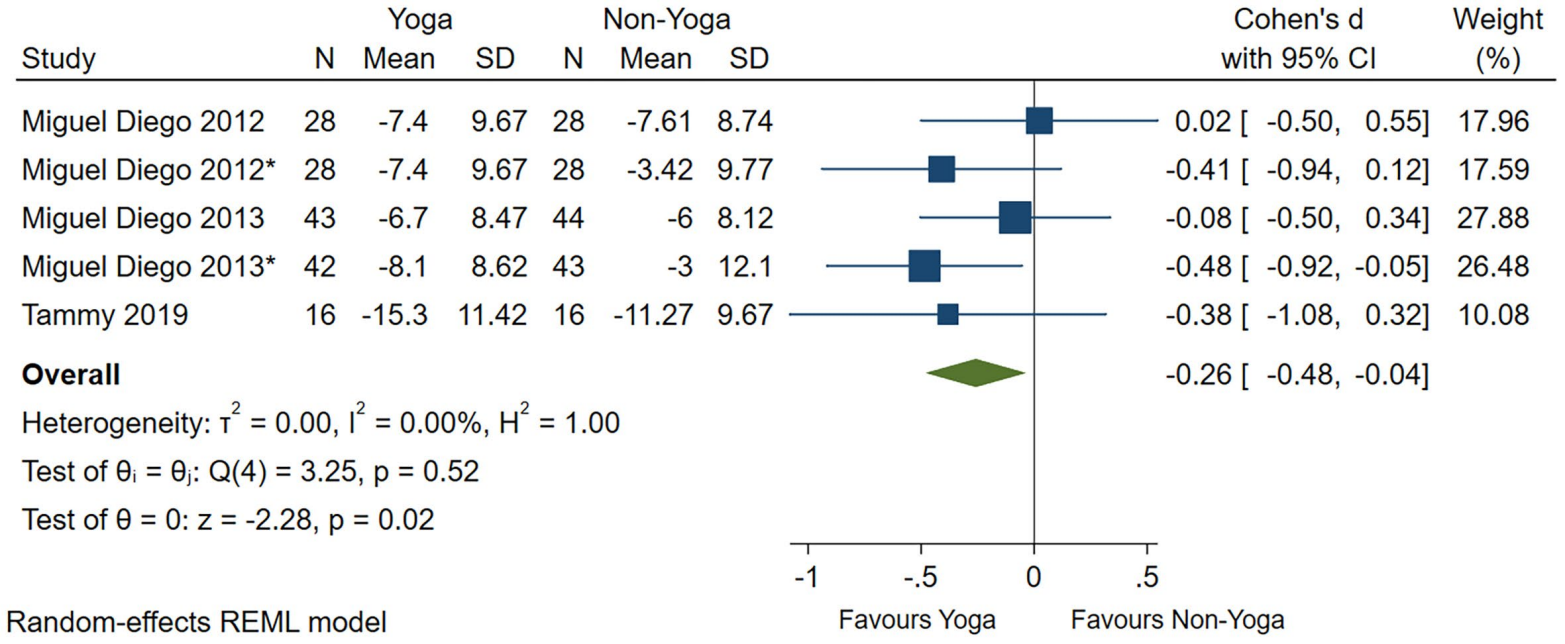
Forest plot of STAI.

**Figure 9:**
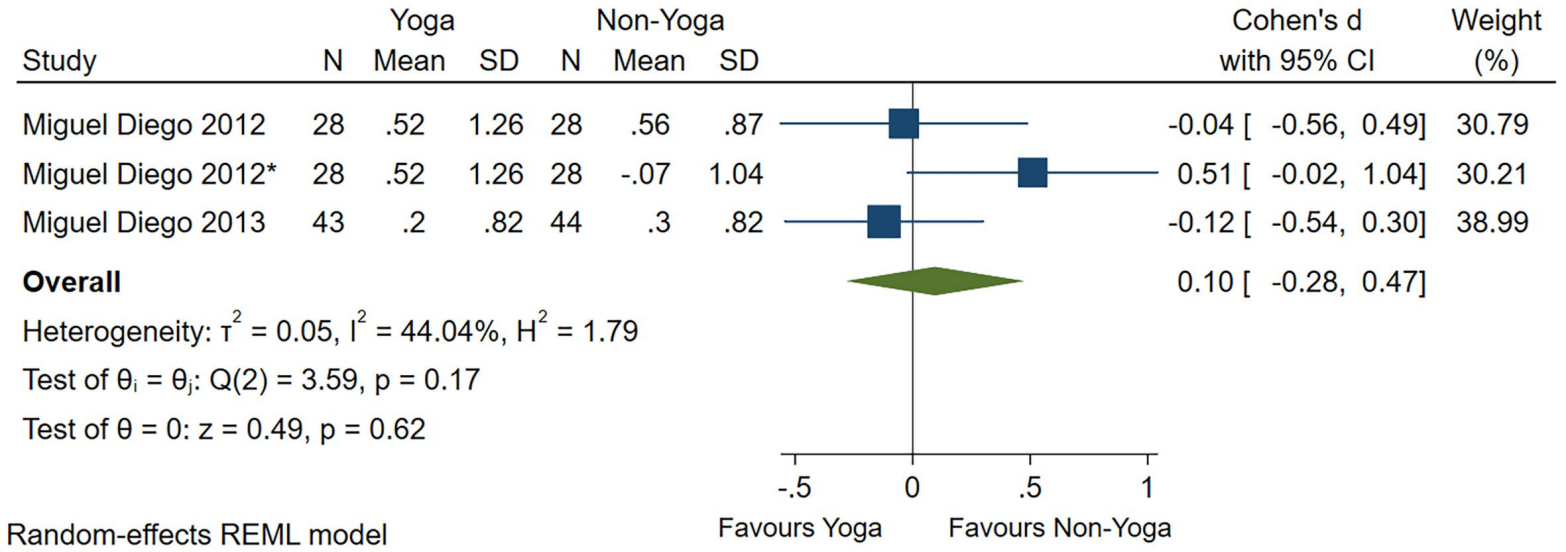
Forest plot of quality of life.

One study had no significant evidence that yoga improved anxiety levels as assessed by the HAMA scale (Cohen’s *d* = −0.49; 95% CI: −1.23 to 0.25; *p* = 0.19; *I*^2^ = 68.48%; *Q* (1) = 3.17, *p* = 0.07) (Figure 7). Four studies assessed by the STAI scale showed that yoga had a small effect on the improvement of anxiety levels (Cohen’s *d* = −0.26; 95% CI: −0.48 to −0.04; *p* = 0.02; heterogeneity: *I*^2^ = 0; *Q* (4) = 3.25, *p* = 0.52) (Figure 8). In two RCTs, there was no significant evidence of improvement in yoga-related quality of life as assessed by relationships (Cohen’s *d* = 0.10; 95% CI: −0.28 to 0.47; *p* = 0.62; heterogeneity: *I*^2^ = 44.04%; *Q* (2) = 3.59, *p* = 0.17) (Figure 9).

In all, one RCT (42) reported that 42% of patients in the yoga group had a 50% reduction in depressive symptoms at a 6-month follow-up compared to 31% in the control group. In addition, yoga had a significant improvement in depressive symptoms, body cortisol concentration, and IL-6 concentration levels but no statistically significant improvement in anxiety levels (Supplementary Figure 1 and Supplementary Table 5).

### 3.5. Subgroup analysis

#### 3.5.1. Subgroup analysis regarding the area of study

**Figure 10:**
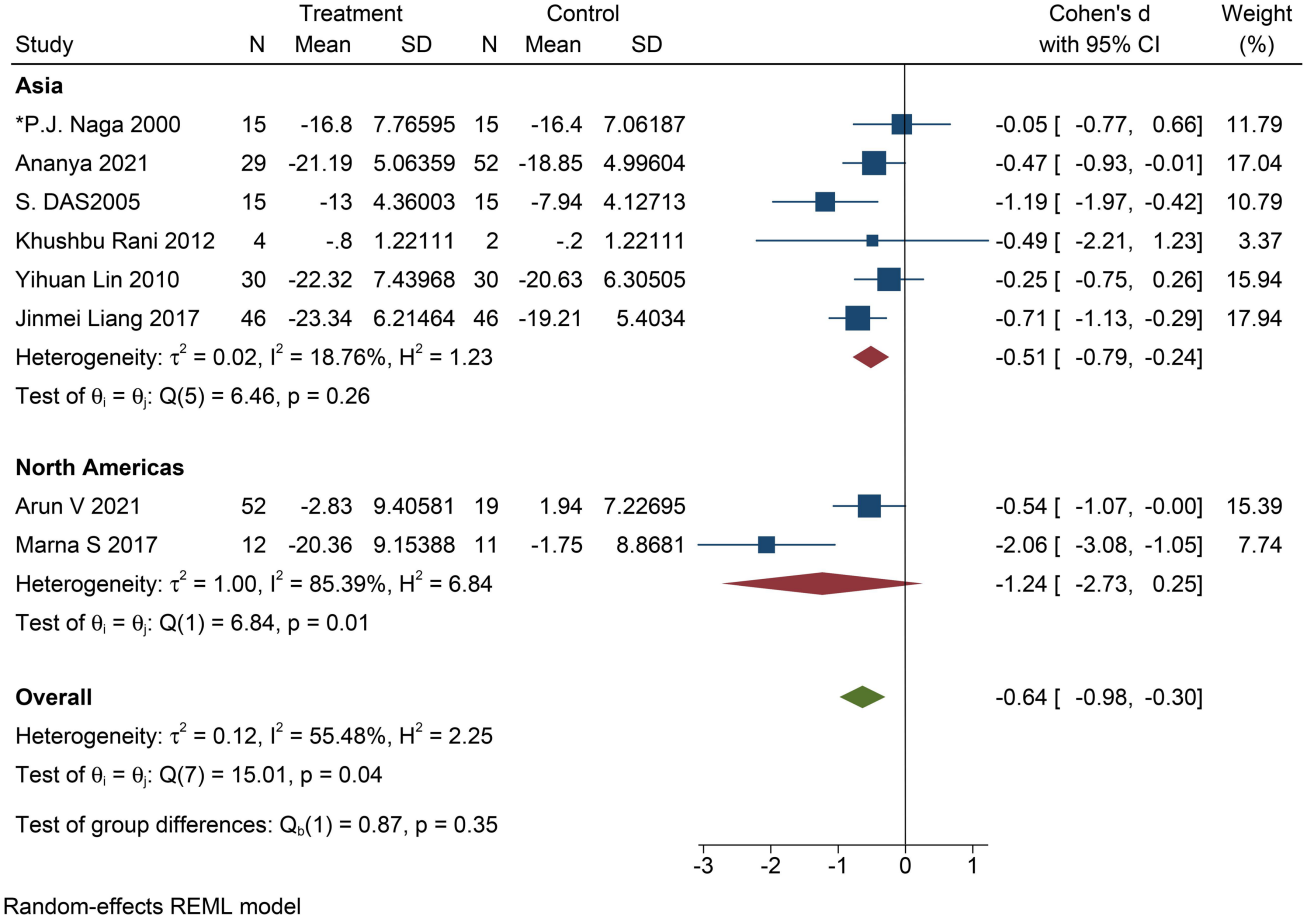
Forest plot of subgroup analysis regarding the region of study.

To determine whether the place where the RCT was conducted was effective in improving the symptoms or degree of depression in patients with MDD, we conducted a subgroup analysis. Six studies were from Asia, and two studies were from the Americas. In the former subgroup: Cohen’s *d* = −0.51; 95% CI: −0.79 to −0.24; *p* < 0.05; *I*^2^ = 18.76%; *Q* (5) = 3.17, *p* = 0.26, and in the latter subgroup: Cohen’s *d* = −1.24; 95% CI: −2.73 to 0.25; *p* = 0.1; *I*^2^ = 85.39%; *Q* (1) = 6.84, *p* = 0.01 (Figure 10). The results showed that studies from Asia were less heterogeneous than those from the Americas and had a moderately positive effect on improving depressive symptoms.

#### 3.5.2. Subgroup analysis regarding intervention type

**Figure 11:**
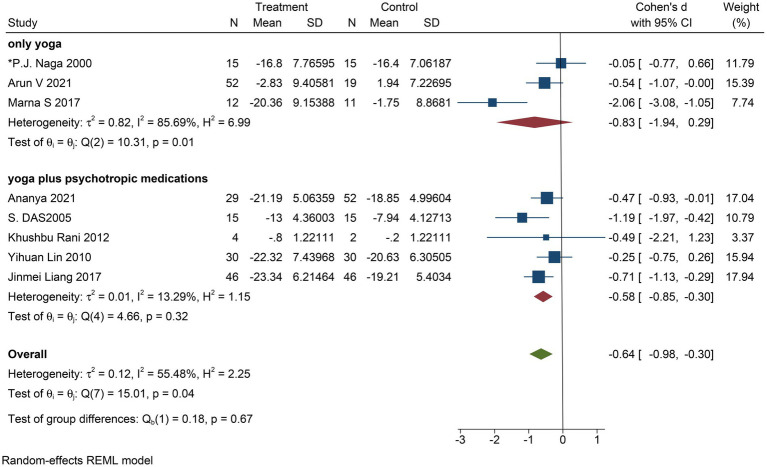
Forest plot of subgroup analysis regarding intervention type.

To determine whether the experimental intervention in a different way for improving the depressive symptoms of patients with severe depression or level effectively, we conducted a subgroup analysis. Group intervention into only yoga and yoga as an aid in the treatment of drugs. There were only three studies with yoga as an intervention and five with yoga and meditation as an intervention. The results show that the intervention included both yoga and meditation (Cohen’s *d* = −0.58; 95% CI: −0.85 to −0.30; *p* = 0.1; *I*^2^ = 13.29%; *Q* (4) = 4.66, *p* = 0.32) compared with the experimental group that received only yoga (Cohen’s *d* = −0.83; 95% CI: −1.94 to 0.29; *p* < 0.0001; *I*^2^ = 85.69%; *Q* (2) = 10.31, *p* = 0.01), depressive symptoms improved significantly (Figure 11).

#### 3.5.3. Subgroup analysis regarding intervention duration

**Figure 12:**
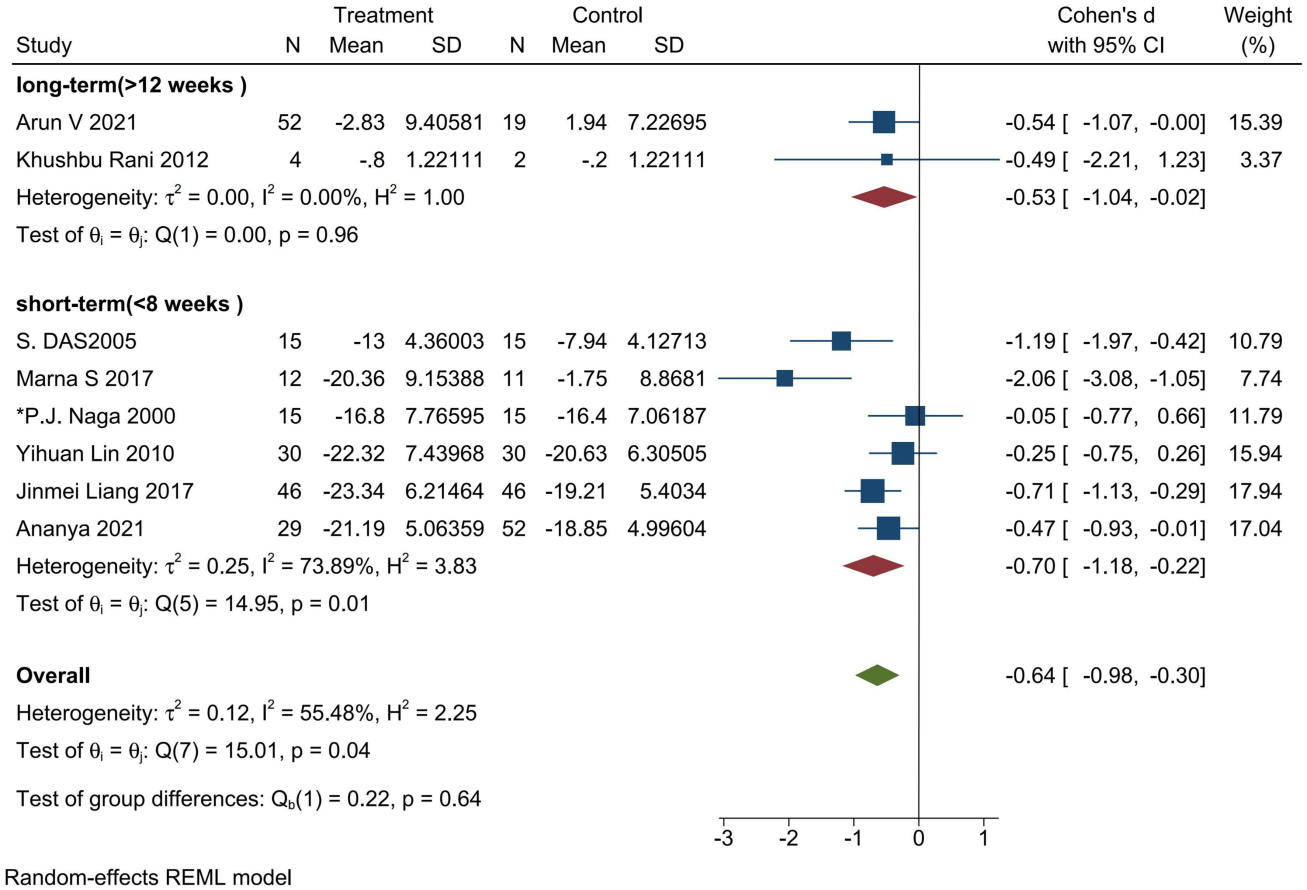
Forest plot of subgroup analysis regarding intervention duration.

To determine whether the duration of the intervention in the trial group was effective in improving depressive symptoms or severity in patients with major depressive disorder, we performed a subgroup analysis. An intervention duration of fewer than 8 weeks was defined as a short-range intervention, and an intervention duration of more than 15 weeks was defined as a long-range intervention. There were six studies on short-term intervention and two studies on long-term intervention. The results showed that the short-term intervention (Cohen’s *d* = −0.70; 95% CI: −1.18 to −0.22; *p* = 0.001; *I*^2^ = 73.89%; *Q* (5) = 14.95, *p* = 0.01) and the long-term intervention (Cohen’s *d* = −0.53; 95% CI: −1.04 to −0.02; *p* = 0.05; *I*^2^ = 0.00%; *Q* (1) = 0.00, *p* = 0.96) had a moderate effect on the improvement of depressive symptoms or severity. In contrast, the impact of the short-term intervention was more significant (Figure 12).

#### 3.5.4. Subgroup analysis regarding intervention frequency

**Figure 13:**
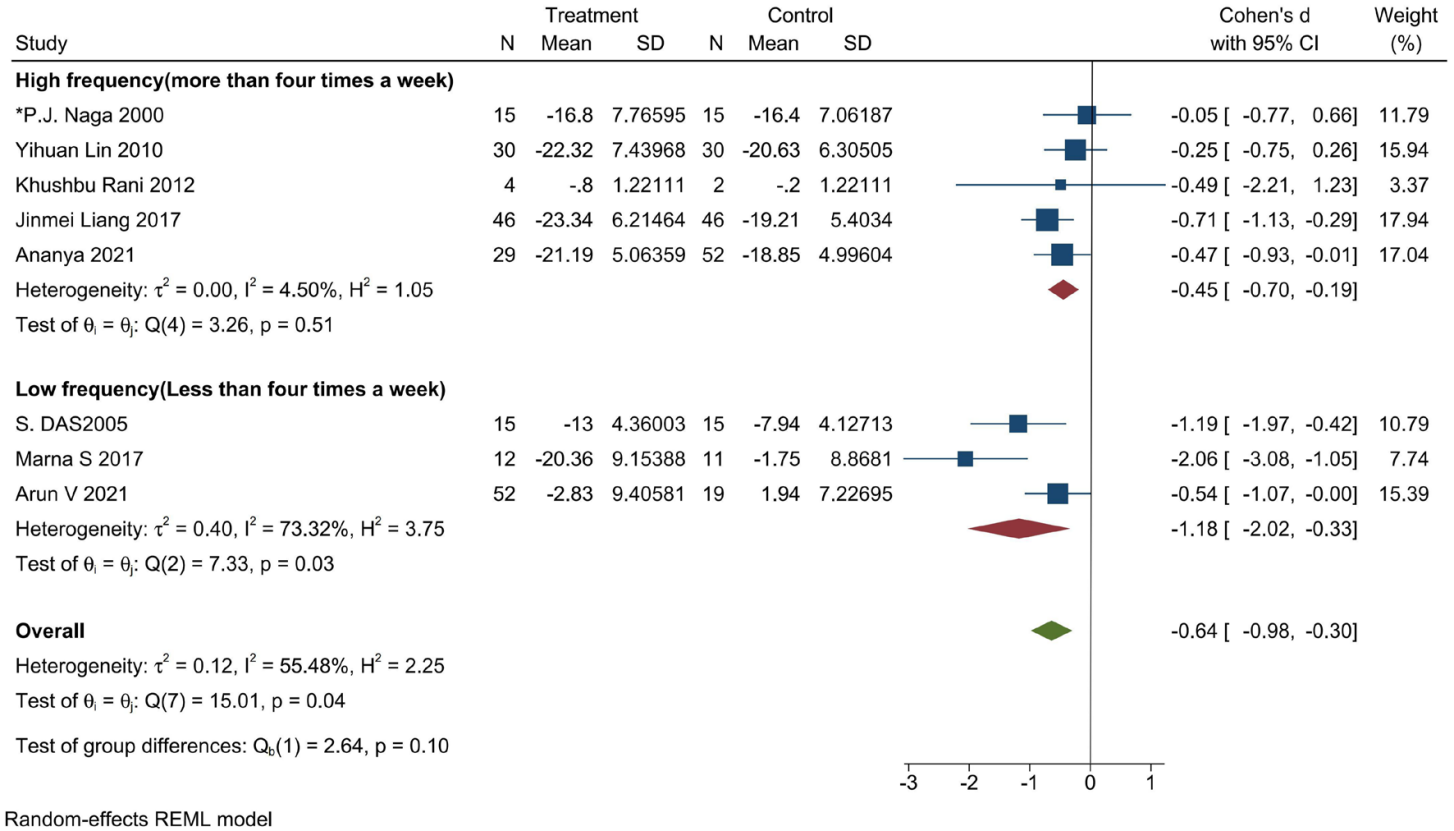
Forest plot of subgroup analysis regarding intervention frequency.

We conducted a subgroup analysis to determine whether the frequency of the intervention in the trial group was effective in improving symptoms or the degree of depression in patients with MDD. Intervention frequency less than four times per week was defined as low-frequency intervention, and intervention frequency more than four times per week was described as high-frequency intervention. There were three studies with low-frequency interventions and five studies with high-frequency interventions. The results showed that the low-frequency intervention (Cohen’s *d* = −1.18; 95% CI: −2.02 to −0.33; *I*^2^ = 73.32%; *Q* (2) = 7.33, *p* = 0.03) and the high-frequency intervention (Cohen’s *d* = −0.45; 95% CI: −0.70 to −0.19; *I*^2^ = 4.50%; *Q* (4) = 3.26, *p* = 0.51) had a moderate effect on the improvement of depressive symptoms or severity. In contrast, the impact of the high-frequency intervention was more significant (Figure 13).

### 3.6. Sensitivity analysis and publication bias

The funnel plot shows some asymmetry (Supplementary Figures 2A–E). The Egger test confirmed that yoga improved MDD without potential publication bias (Supplementary Table 4).

The results of the sensitivity analysis showed that on the sensitivity analysis chart of HAMD, the combined effect was −0.63, 95% CI (−0.96, −0.30), and the combined effect size did not change significantly after excluding any study (Supplementary Figure 4A). This indicates that the meta-analysis results are relatively stable. The sensitivity analysis showed that on the sensitivity analysis chart of BDI-II, the combined effect was −0.60, 95% CI (−1.00, −0.21). The combined effect size did not change significantly after excluding any study (Supplementary Figure 4B). This indicates that the results of the meta-analysis are stable. The sensitivity analysis showed that on the sensitivity analysis chart of STAI, the combined effect was −0.24, 95% CI (−0.48, −0.01), and the combined effect size did not change significantly after excluding any study (Supplementary Figure 4C). This indicates that the results of this meta-analysis are stable. The sensitivity analysis showed that on the sensitivity analysis chart of the relationship, the combined effect was 0.10, 95% CI (−0.28, 0.47), and the combined effect size did not change significantly after excluding any study (Supplementary Figure 4D). This indicates that the results of the meta-analysis are stable. The results of the sensitivity analysis showed that on the sensitivity analysis chart of STAIX, the combined effect was −0.32, 95% CI (−0.60, −0.04), and the combined effect size did not change significantly after excluding any study (Supplementary Figure 4E). This indicates that the results of the meta-analysis are stable.

## 4. Discussion

This study systematically evaluates the effectiveness of yoga in improving depressive symptoms in patients with MDD. It validates the evidence for the efficacy of yoga for short-term improvements in depressive symptoms and anxiety levels in patients with MDD. Patients diagnosed with MDD in the included studies were recruited from inpatient and outpatient psychiatric services in North America, India, Europe, and China. The participants had the general population, prenatal women, postpartum women, and students, with the majority of participants being female and with a mean age of 32.39 years. Subjects in all nine studies were female, thus, the evidence appears to be more applicable to female patients. This study still has some limitations. First, a moderate number of studies were included in this article, but some of the included articles were published long ago and were of low quality. Second, we did not conduct a subgroup analysis to compare the differences between yoga alone and yoga as an adjunct to psychotherapy. Third, we could not conclude the optimal duration and frequency of exercise for yoga to improve depressive symptoms.

The second subgroup analysis showed that yoga as an adjunct combination with medication was more effective in improving severe depression alone. With the subgroup analysis of the study (60) result is consistent. As well as in the review (61) also mentioned yoga as a supplement to antipsychotic medications to help reduce psychopathology and improve socio-occupational functioning. The third subgroup analysis showed that the effect of randomized controlled trials for short yoga intervention (<8 weeks) was more significant, indicating that brief yoga intervention can improve symptoms of severe depression in a short period. Still, some studies suggested that yoga must be performed for a certain period to obtain the maximum benefit. Most studies described here had a maximum intervention period of 6 months. Therefore, the optimal course of yoga intervention is still an issue worth discussing. The fourth subgroup analysis showed that a weekly high-frequency yoga intervention significantly improved severe depression. However, it is unclear how the various postures used in these studies resulted in therapeutic effects, possibly involving concentration and control of breathing (62).

The pathogenesis of MDD has not been elucidated and is now thought to be caused by biological, genetic, environmental, and psychosocial factors. Early on, MDD was supposed to be associated with abnormalities in monoamine neurotransmitters such as serotonin, norepinephrine, 5-HT, and dopamine. Thus, the mechanisms underlying many clinical treatments for depression were based on this. In contrast, recent theories suggest that it is related to more complex neuromodulator systems and neural circuits. Some scholars suggest that psychological stress, inflammatory cytokines, and hypothalamic–pituitary–adrenal axis dysfunction may trigger depression (63–65). In addition, it has also been recommended that MDD results from disruption of homeostatic mechanisms controlling synaptic plasticity, including reduced synaptic connections between the frontal lobe and other brain regions, neurotrophic and altered dendritic numbers, and increased connectivity within the DMN (66–68). In clinical practice, less than 40% of patients with MDD achieve remission with initial treatment, and approximately 20–30% of patients with MDD do not respond adequately to standard therapy. In one clinical trial, antidepressants had substantial antidepressant effects that exceeded the placebo effect in approximately 15% of patients with MDD compared with placebo in a clinical trial (69). A study conducted in Denmark describing treatment patterns in patients with refractory depression (TRD) and MDD showed that 15% of patients with MDD met the diagnostic criteria for TRD and that treatment for this group of patients was haphazard and did not follow the first treatment guidelines for MDD, probably because of the lack of specific procedures (70).

The number of studies addressing yoga to improve symptoms of depression has been gradually increasing in recent years. A recent Cochrane review and meta-analysis found (71) that exercise was moderately effective in reducing depressive symptoms in depressed adults compared to controls [standardized mean difference (SMD) = −0.62 (95% CI: −0.81 to −0.42)], and that yoga as a soothing exercise and its-based interventions have good therapeutic research. The research has shown that yoga-based interventions are a safe and effective treatment for depression as well as other psychiatric disorders, and a recent meta-analysis (18) whose included 13 studies with conditions such as depression, posttraumatic stress, schizophrenia, anxiety, alcohol dependence, and bipolar disorder, this meta found that yoga provided more relief from depressive symptoms than did waiting for conventional treatment and attentional control (standardized mean difference = 0.41; 95% CI −0.65 to −0.17; *p* < 0.001). Depressive symptoms were significantly reduced with the higher frequency of weekly yoga practice (*β* = −0.44, *p* < 0.01), and yoga was more acceptable and more accessible to adhere to than electroconvulsive therapy. Yoga is an exercise for both young and old, and of the 34 RCTs included in this article, nine were conducted with female subjects. The participants in three of the studies were pregnant, showing that yoga is effective in improving depression in women with perinatal depression (72), postpartum depression (73), and adolescent children (74). More recently, studies have demonstrated the effectiveness of yoga and mindfulness in the treatment of refractory depression and the prevention of relapse (75). In another study, yoga was evaluated for adverse effects, safety, and potential effects on suicidal ideation. Thirty-two patients with MDD were randomized to a high-dose group (HDG) and a low-dose group (LDG) for 12 weeks of intervention. The HDG group consisted of three 90-min yoga sessions per week, while the LDG group consisted of two 90-min yoga sessions per week for 12 weeks of intervention. A total of 30 participants completed the intervention. The most common adverse effect was musculoskeletal soreness, which was reported by 16 of the 30 participants, but all resolved throughout the study; other than that, no other serious adverse effects were reported. No patients withdrew midway through the study due to skeletal pain or other adverse effects, and no risk of worsening psychiatric symptoms was observed, suggesting that the side effects of yoga are incredibly mild and well tolerated by patients with MDD while having a high-safety profile (19). There are nine (19, 39–43, 47, 48, 51) studies that evaluated adverse reactions, and no serious adverse events were found. Among them, mild musculoskeletal soreness occurred in four (19, 39, 41, 47) studies, but all were relieved during the study, and no subjects were withdrawn. In total, two (19, 41) studies were assessed weekly using adverse event forms filled out by subjects and assessed using adverse event forms at the fourth, eighth, and 12th week (the end point of intervention) of the intervention by physicians or psychologists. Overall, one (42) study was assessed every 3 weeks, while the other six did not mention the specific evaluation method. A previous review (76) looked at the safety of yoga interventions and the frequency of adverse events from randomized controlled trials and found that the frequency and severity of yoga-related adverse events were comparable to that associated with physical activity or usual care. However, yoga may be associated with more frequent non-serious adverse events than psychological or educational interventions (i.e., interventions that typically do not involve physical activity). Consistent with this article, limited evidence shows that yoga is still a safe intervention, with only a tiny proportion of serious adverse events occurring. Therefore, it is necessary to report comprehensive and detailed safety-related data in randomized controlled trials with yoga as the intervention method in the future, including evaluating the time point when adverse events occurred and the frequency of all adverse events. In addition, reporting adverse events should follow international guidelines (77), and further studies with larger sample sizes are needed.

For noninvasive brain stimulation, a hot topic in recent years, in a recent meta-analysis of the variability of the treatment effect of noninvasive brain stimulation, no significant improvement in the treatment effect of noninvasive stimulation compared to sham stimulation was found in the depression group of patients, and individual differences in the influencing factors are essential. Thus, personalizing treatment remains an open question (78). Although most patients with MDD can benefit from established treatments, some symptoms remain resistant to treatment. For patients suffering from major depressive disorder, new therapies are urgently needed. Studies have shown that depression is often highly comorbid with other psychiatric disorders, such as anxiety disorders (79). A total of 65% of patients with depression also have physical comorbidities, often including obesity, type 2 diabetes, metabolic syndrome, and cardiovascular disease (80). In patients with MDD, physical inactivity and a sedentary lifestyle are strongly associated with depressive symptoms. Current international guidelines for treating mental disorders now recommend physical activity-based interventions as part of routine psychiatric treatment (81). Although these recommendations exist, their actual application to treatment is not easy (82), which should also receive adequate attention.

## 5. Conclusion

Yoga presents a non-negligible clinical effect in improving depressive symptoms during treatment and has a safe and wide patient acceptance. RCTs with larger sample sizes and better clinical method designs are still needed to evaluate the level of evidence.

## Data availability statement

The original contributions presented in the study are included in the article/Supplementary material, further inquiries can be directed to the corresponding author.

## Author contributions

YW and DY collected the data, prepared the manuscript, revised the manuscript, carried out the supervision, checked, verified all data, and finalized the approval of the manuscript. YW created figures and conducted the quality assessment. JY designed and supervised the work. All authors contributed to the manuscript and approved the submitted version.

## Conflict of interest

The authors declare that the research was conducted in the absence of any commercial or financial relationships that could be construed as a potential conflict of interest.

## Publisher’s note

All claims expressed in this article are solely those of the authors and do not necessarily represent those of their affiliated organizations, or those of the publisher, the editors and the reviewers. Any product that may be evaluated in this article, or claim that may be made by its manufacturer, is not guaranteed or endorsed by the publisher.
