# Supplementary material for: Effectiveness of yoga for major depressive disorder: A systematic review and meta-analysis
Source: Front Psychiatry. 2023 Mar 23;14:1138205. doi: 10.3389/fpsyt.2023.1138205 (PMC10077871; doi:10.3389/fpsyt.2023.1138205)
Supplement: Supplementary file 1 [file Data_Sheet_1.docx]

Supplementary Material

**Effectiveness of Yoga for Major Depressive Disorder: A Systematic Review and Meta-Analysis**

Jianli Yang*, Yufei Wu, Danni Yan

*** Correspondence:** Jianli Yang: adyy005@163.com

# Supplementary Figures and Tables

## Supplementary Figures 1

**
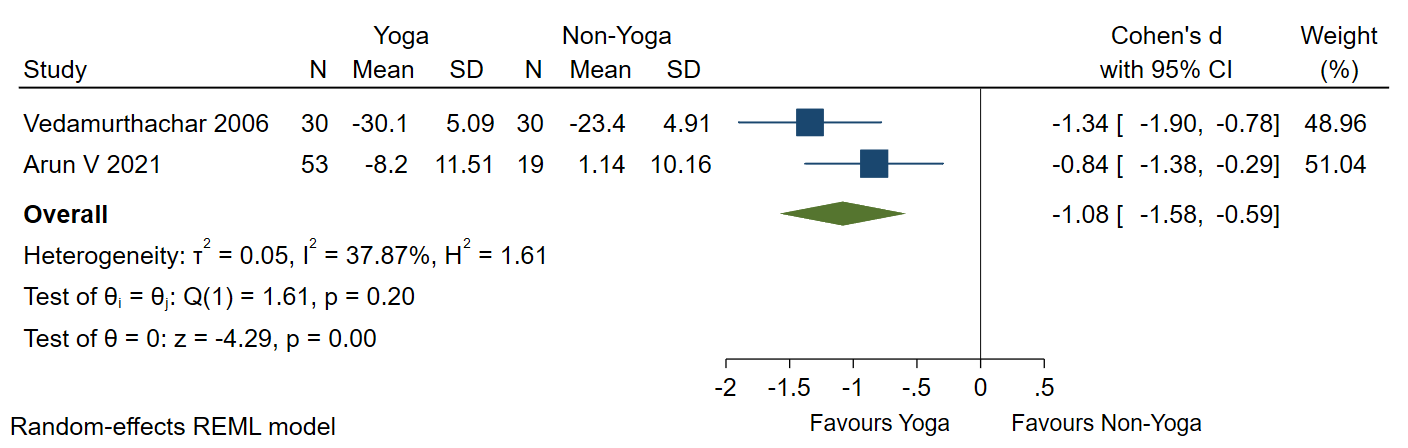
**

**(A)**


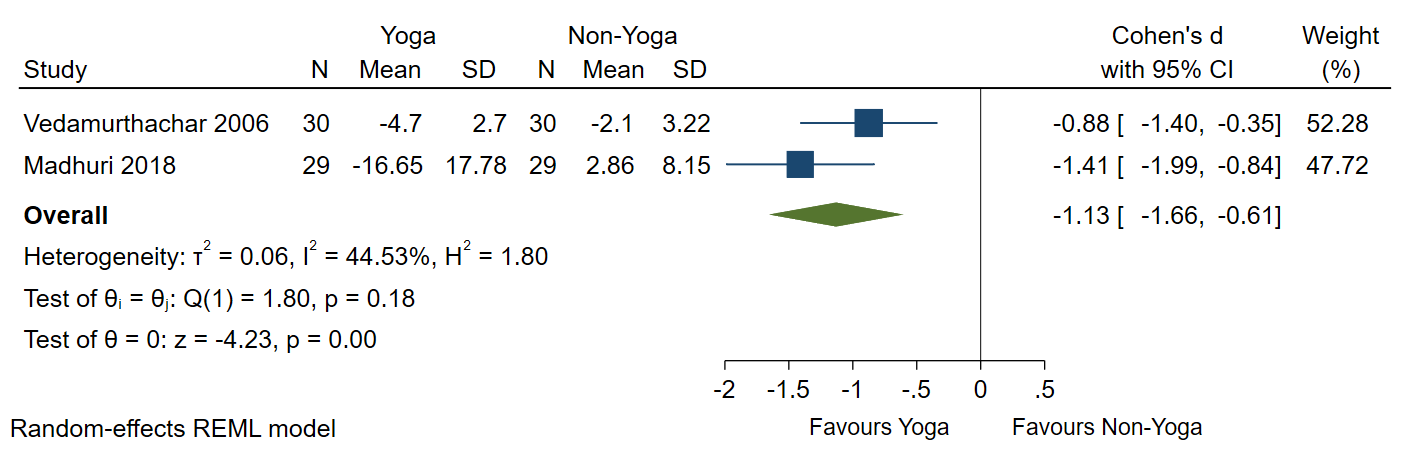
 **(B)**


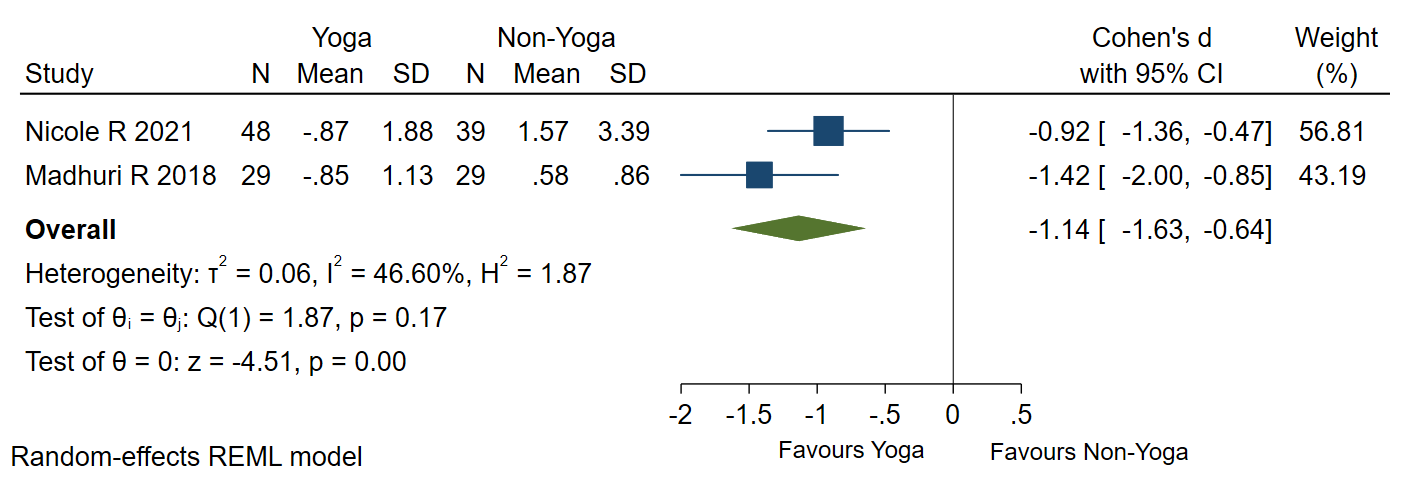


**(C)**


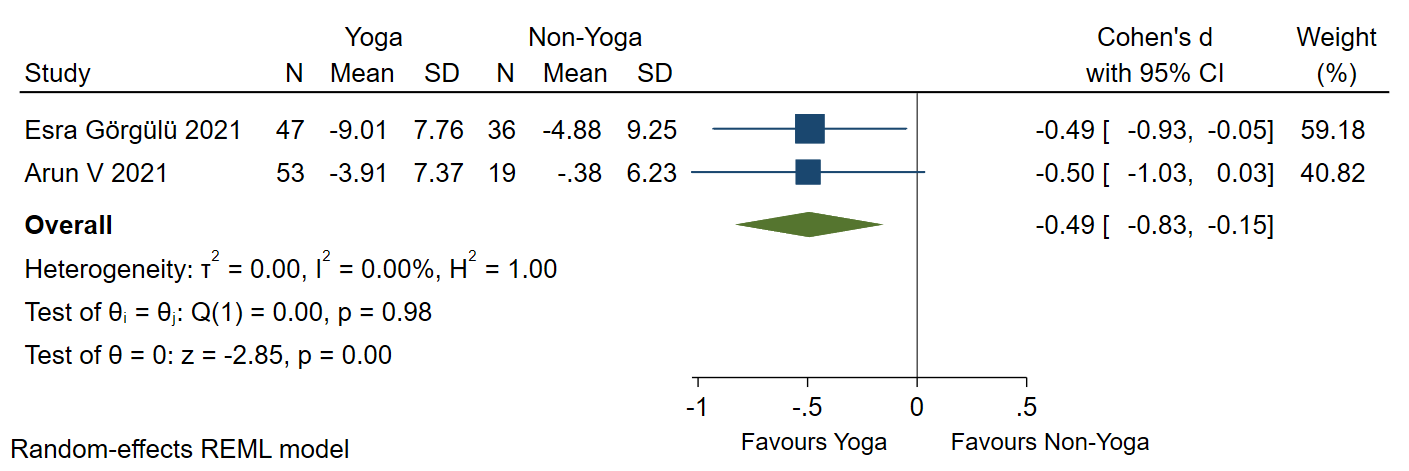


**(D)**


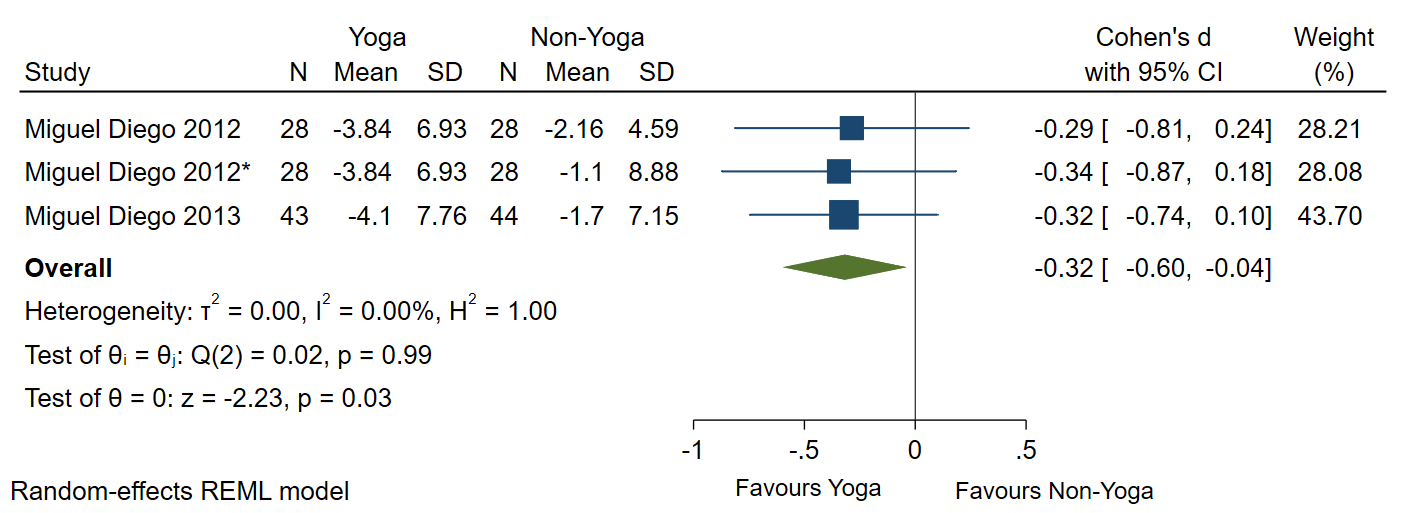


**(E)**

**Supplementary Figure 1.**  **(A) The forest plot of BDI; (B) The forest plot of Cortisol; (C) The forest plot of IL-6; (D) The forest of MADRS; (E) The forest of STAXI;**

## Supplementary Figures 2


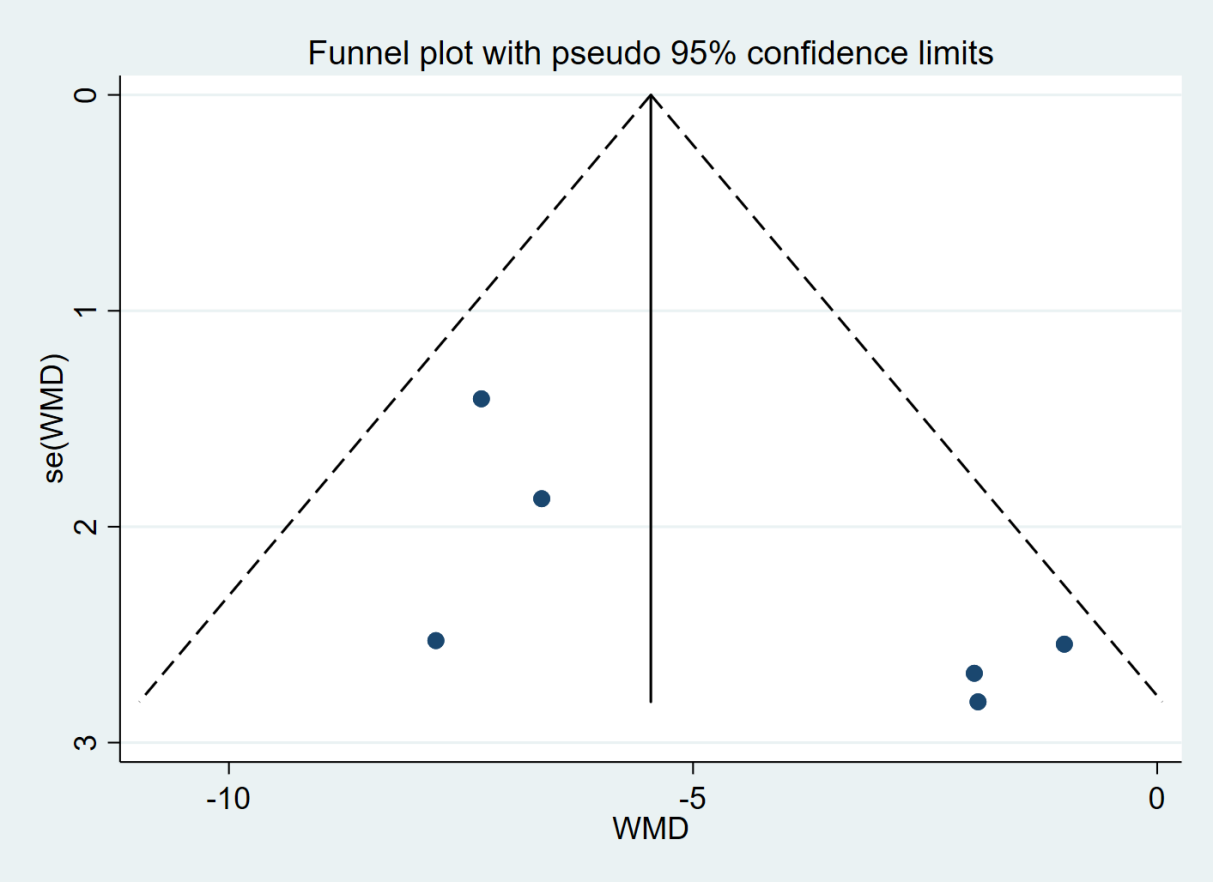


**(A)**


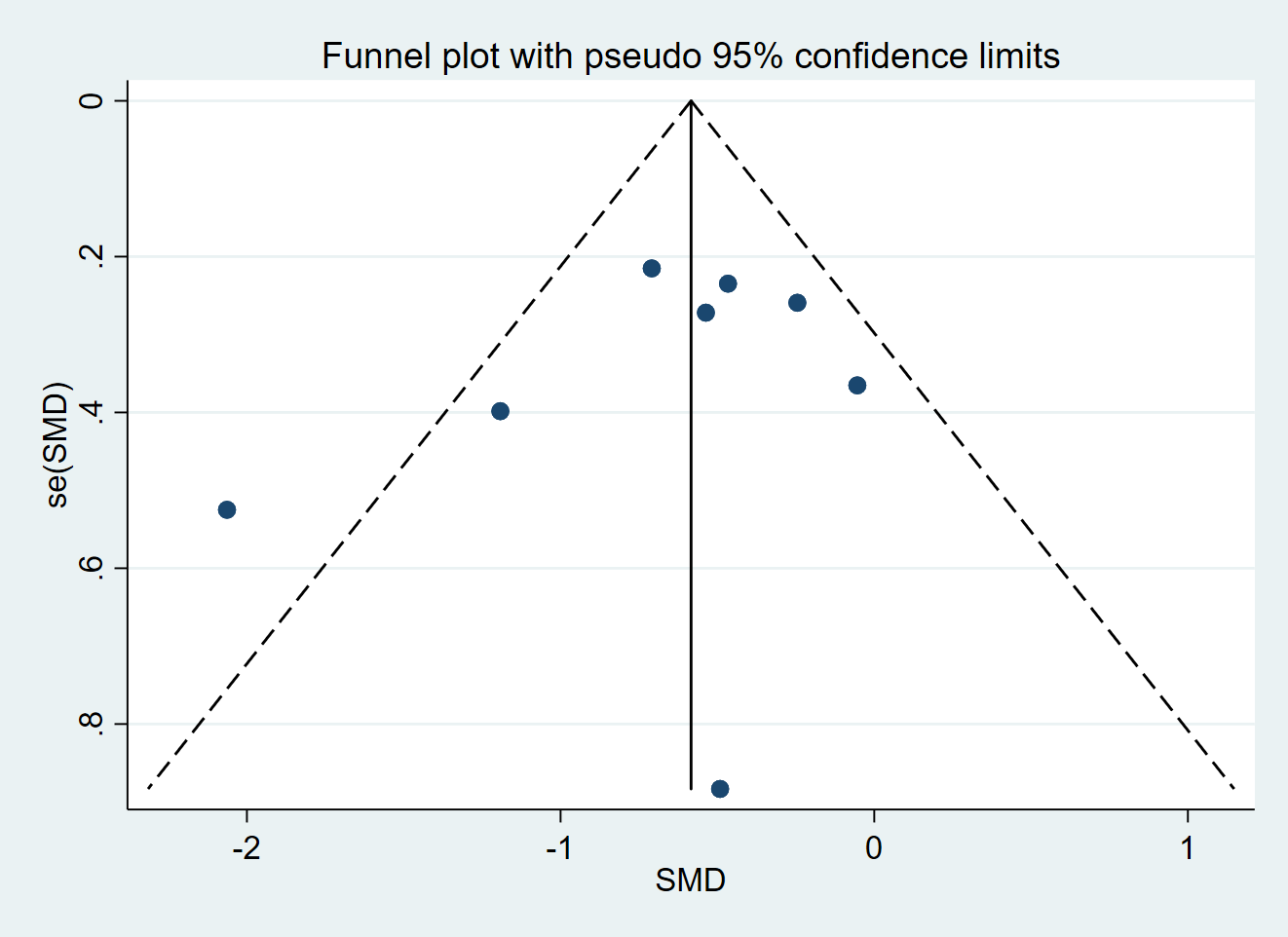


**(B)**


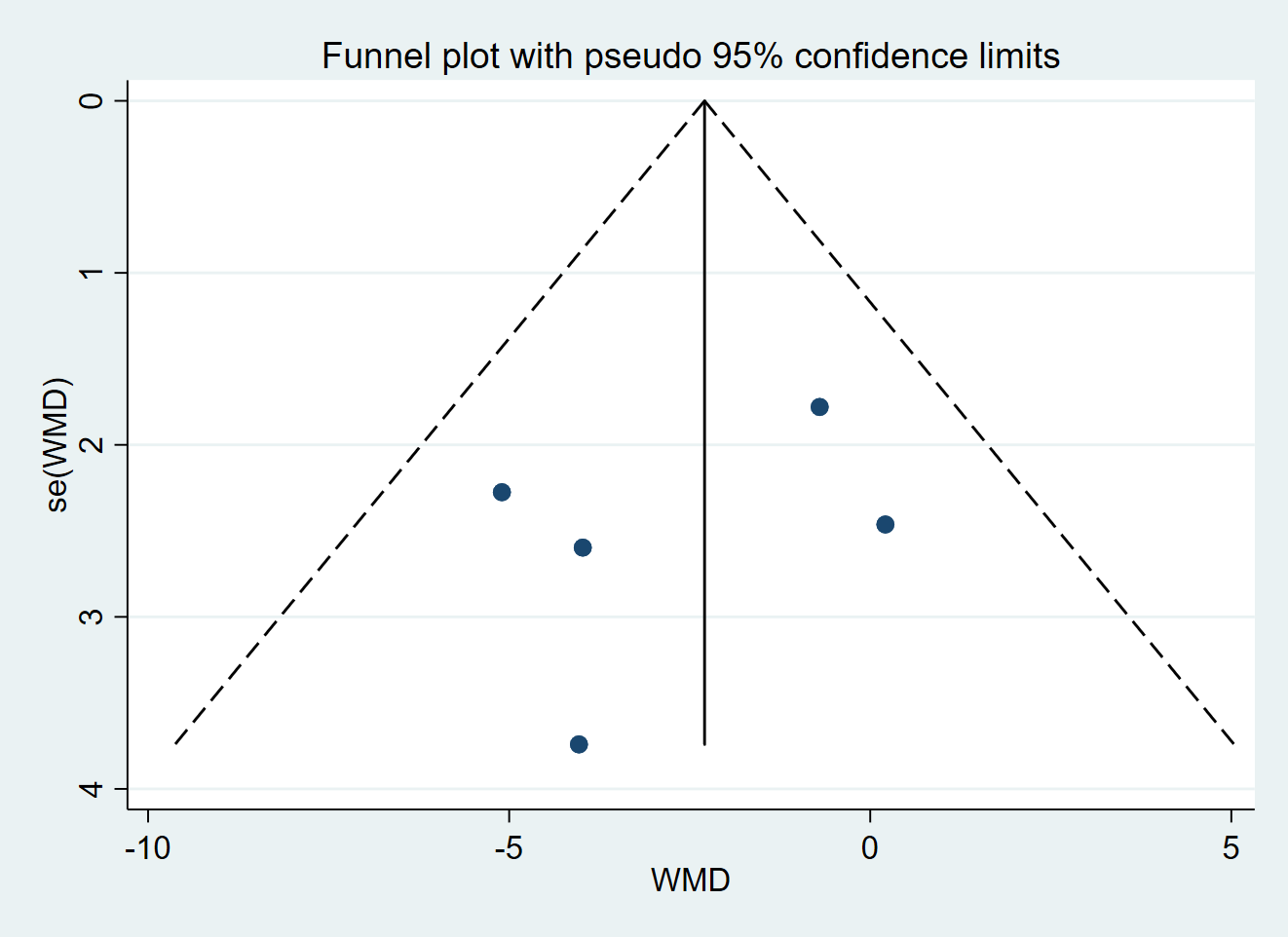


**(C)**


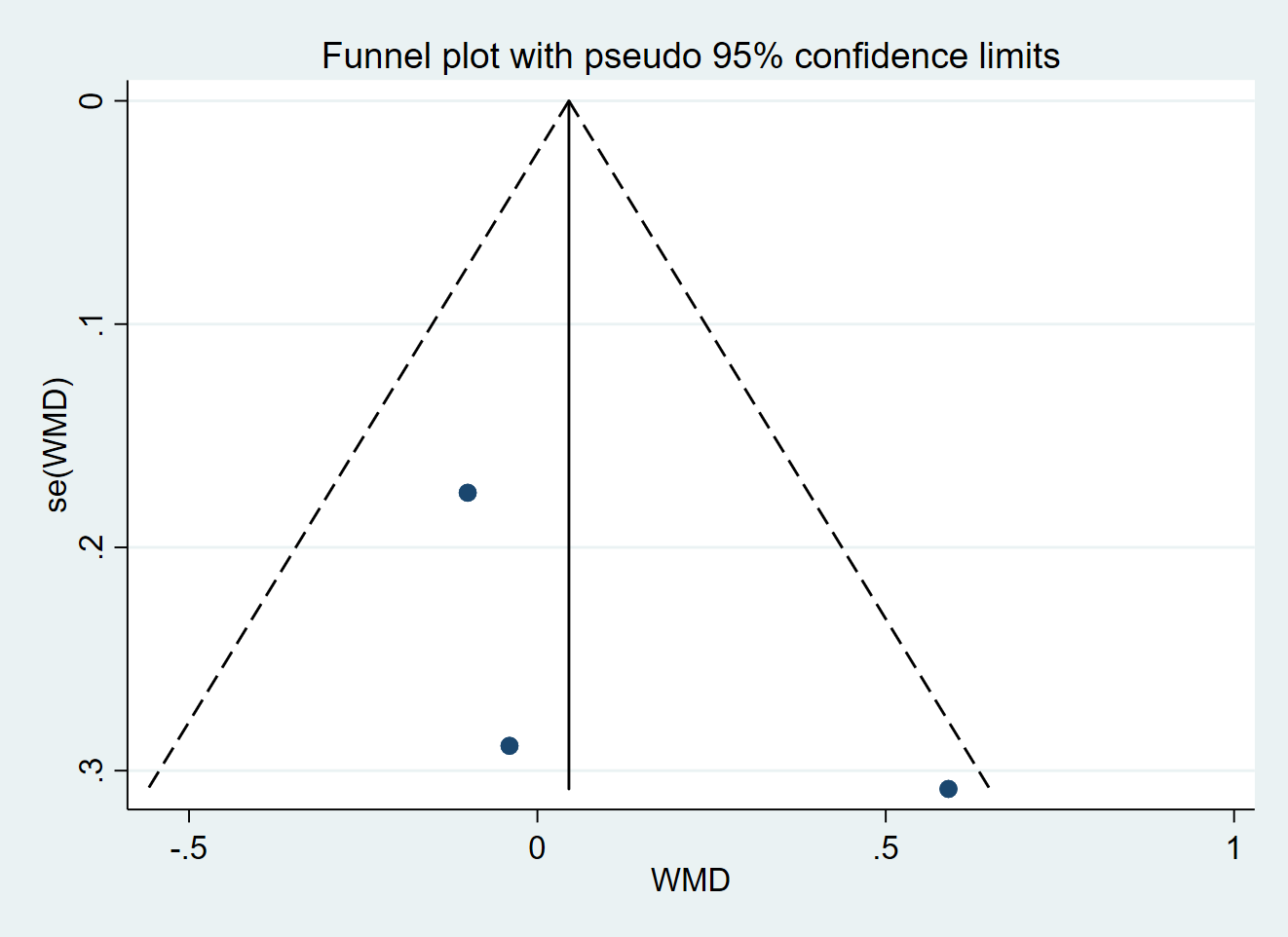


**(D)**


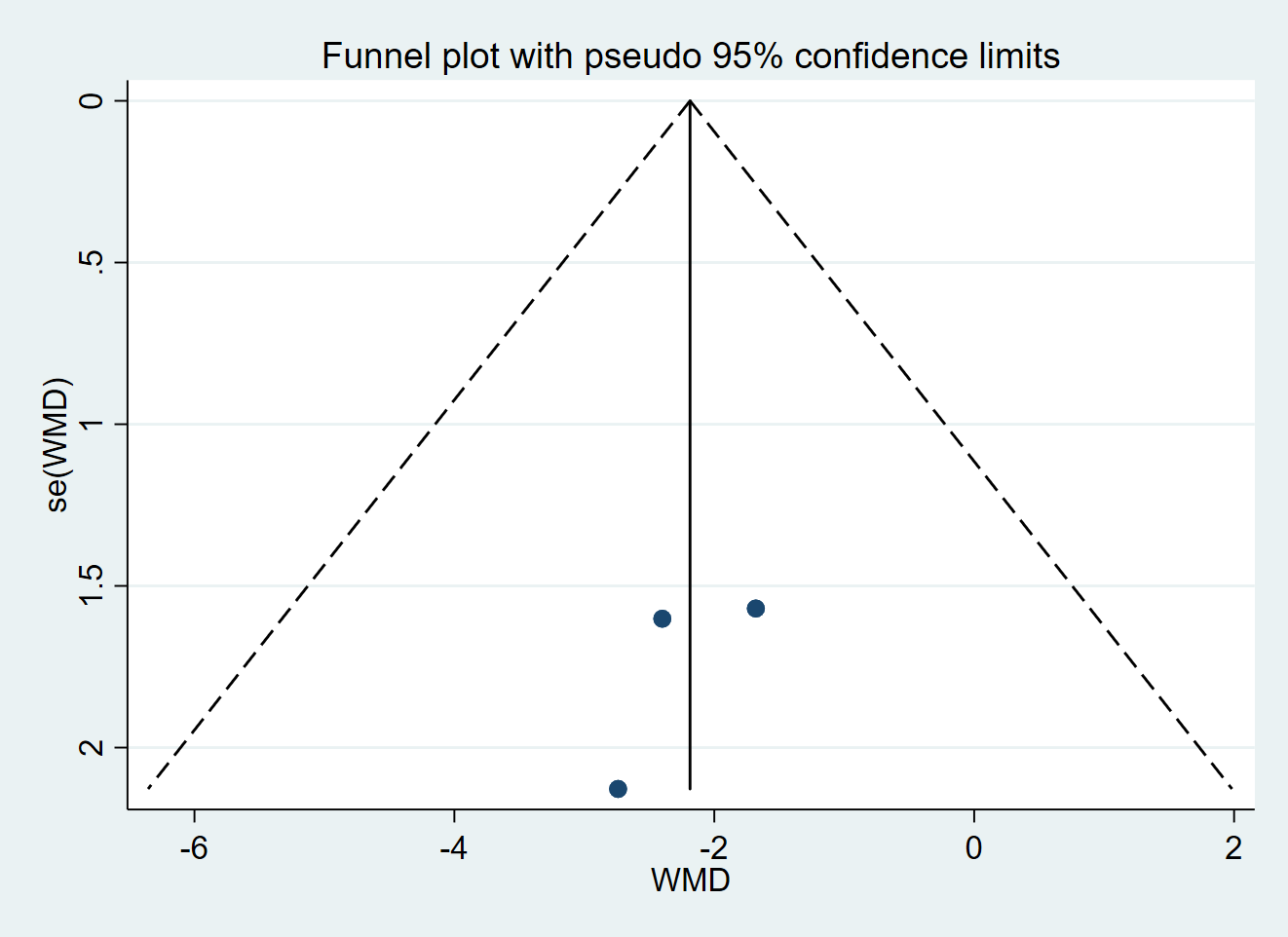


**(E)**

**Supplementary Figure 2.** **(A)The funnel plot of BDI-Ⅱ; (B) The funnel plot of HAMD; (C)The funnel plot of STAI; (D) The funnel plot of Relationships; (E) The funnel plot of STAXI;**

The funnel plot is a method to visually identify publication bias. This method takes the effect quantity as the abscissa, and the standard error is the ordinate. The dashed line perpendicular to the horizontal axis indicates the combined effect size, and ideally, the studies should be evenly distributed on both sides. The larger the sample size, the smaller the standard error, the higher the accuracy, and the more concentrated the research is in the middle and upper part of the graph; The sample size is small, the standard error is large, the accuracy is low, and the distribution is more scattered. Therefore, the ideal funnel diagram is that large sample studies are concentrated at the top and small sample studies are scattered at the bottom, which looks like an inverted funnel. If there is publication bias, the funnel map will have missing corners. At the same time, it is worth noting that when there are too few included studies, it is difficult to judge the symmetry of the funnel diagram, so at least 10 or more included studies are included. In addition, the judgment of funnel symmetry is relatively subjective, and different people may interpret different results. Therefore, the quantitative detection of publication bias, namely egger's test and beck's test, is introduced.

## Supplementary Figure 3

**
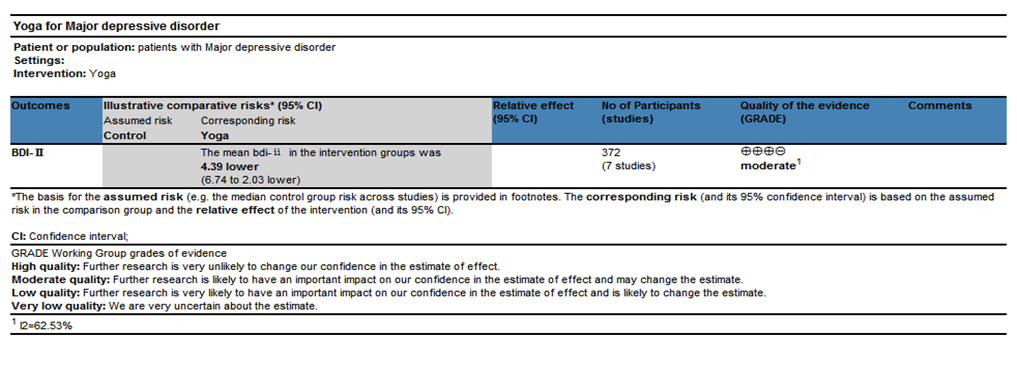
**

**(A)**

**
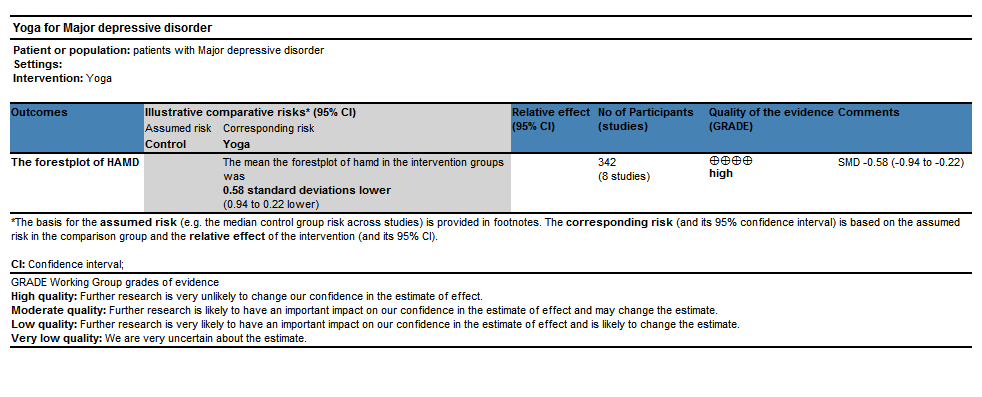
**

**(B)**

**
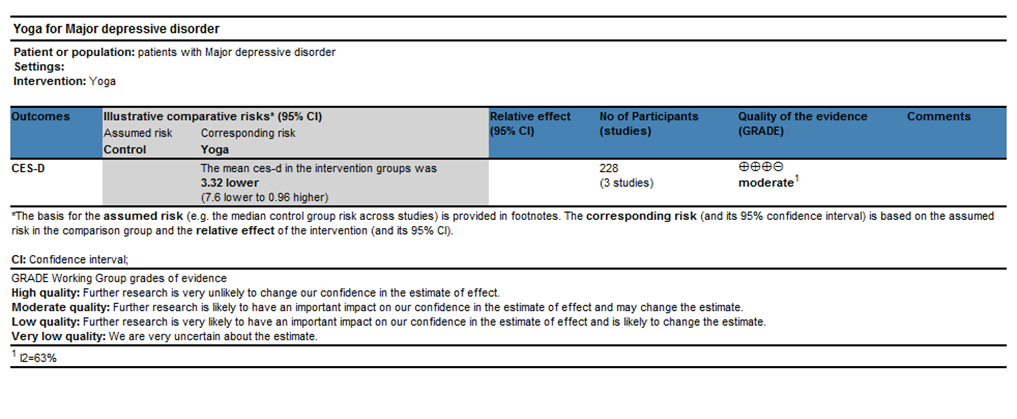
**

**(C)**

**
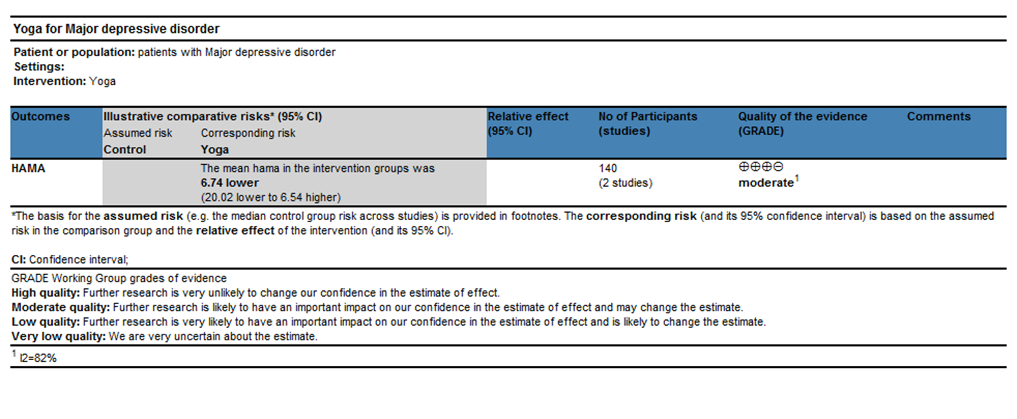
**

**(D)**


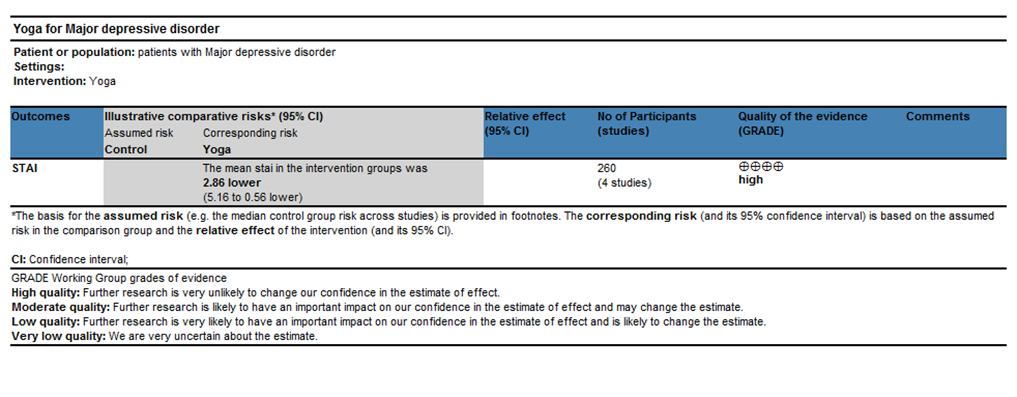


**(E)**

**
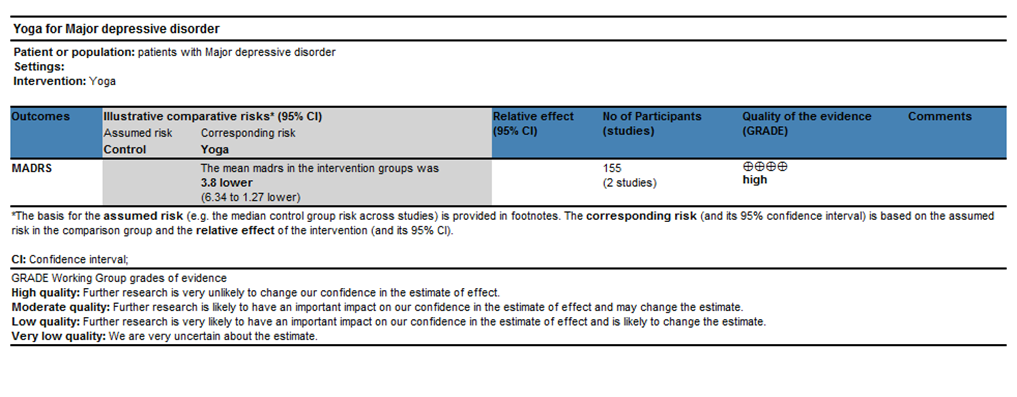
**

**(F)**

**Supplementary Figure 3.** **(A) Rating chart of research evidence assess by BDI-Ⅱ(B)Rating chart of research evidence assess by HAMD;(C) Rating chart of research evidence assess by CES-D;(D) Rating chart of research evidence assess by HAMA;(E) Rating chart of research evidence assess by STAI;(F) Rating chart of research evidence assess by MADRS**

These figures show that: The quality of evidence of BDI-II is moderate; The evidence quality level of HAMD is high; The quality of evidence of CES-D is moderate; HAMA's evidence quality level is moderate; The evidence quality level of STAI is high; The quality of evidence of MADRS is high.

##
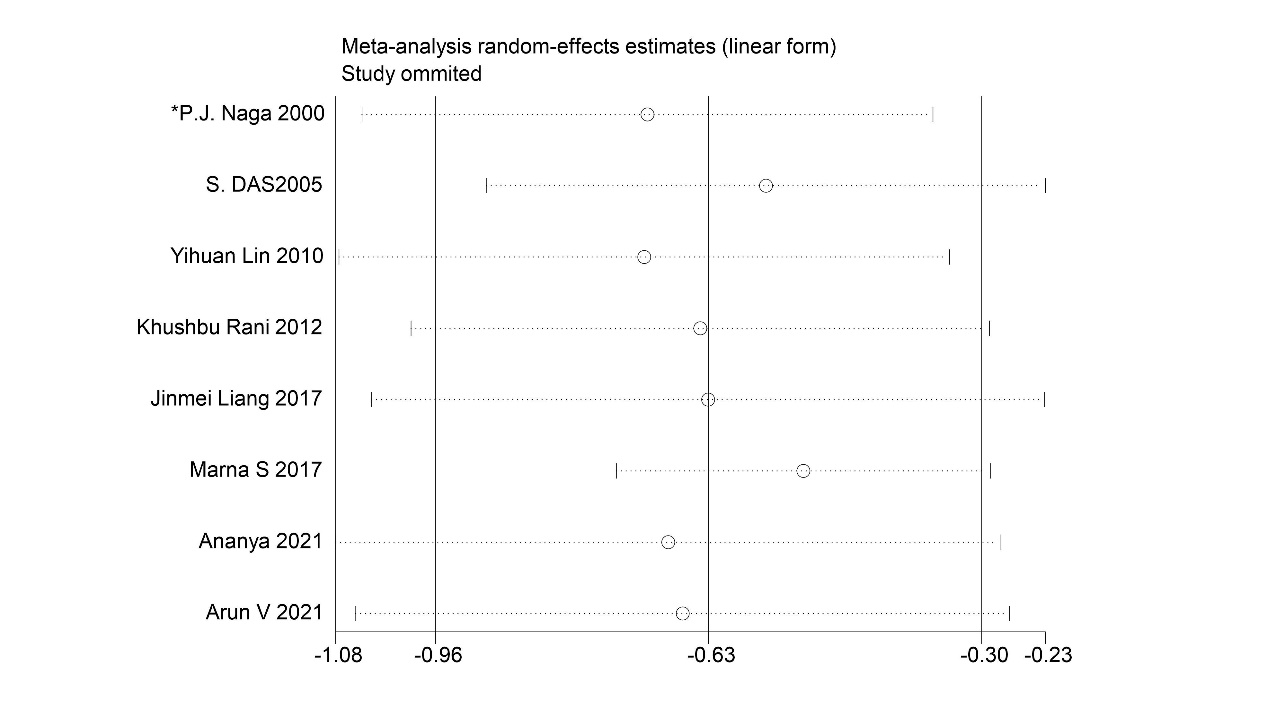
Supplementary Figure 4

**(A)**

**
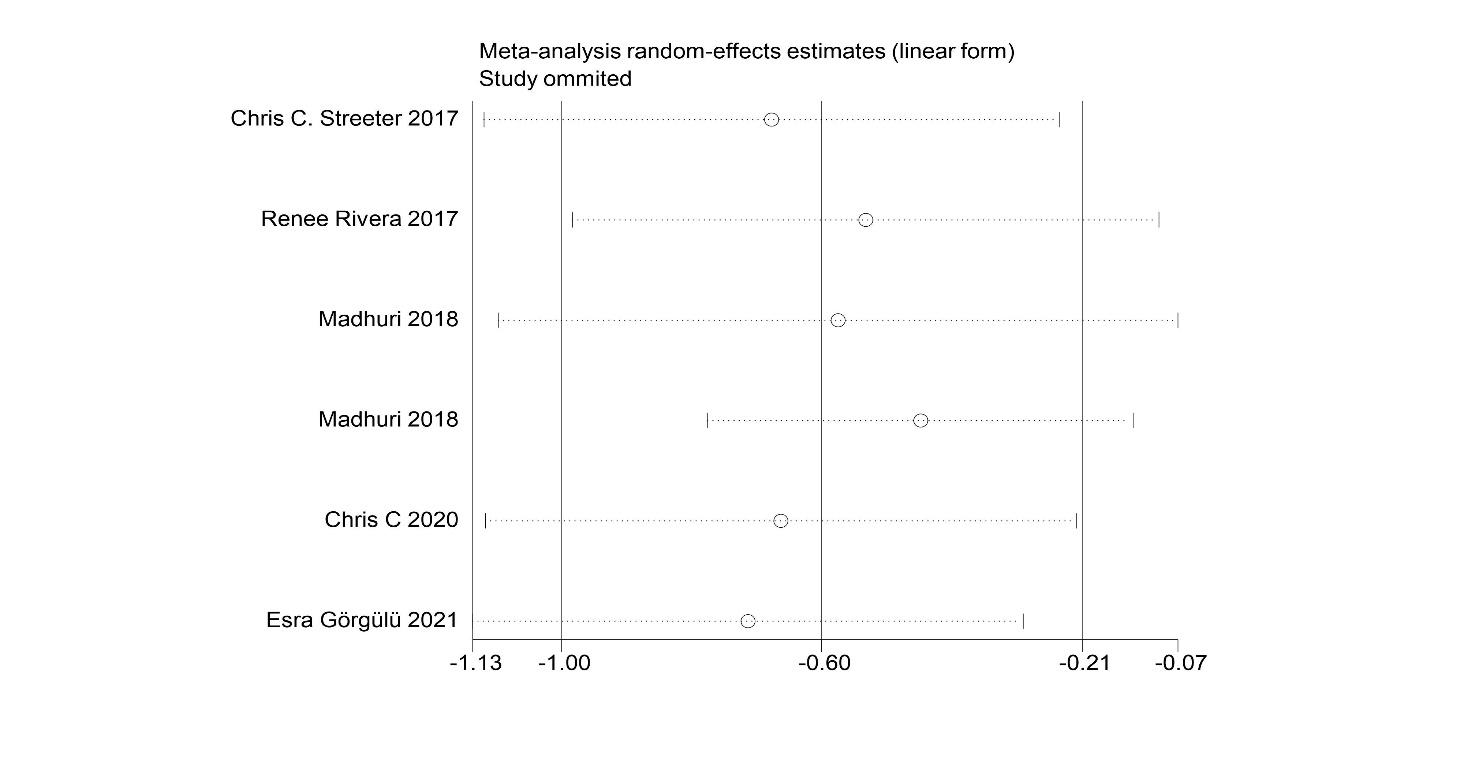
**

**(B)**

**
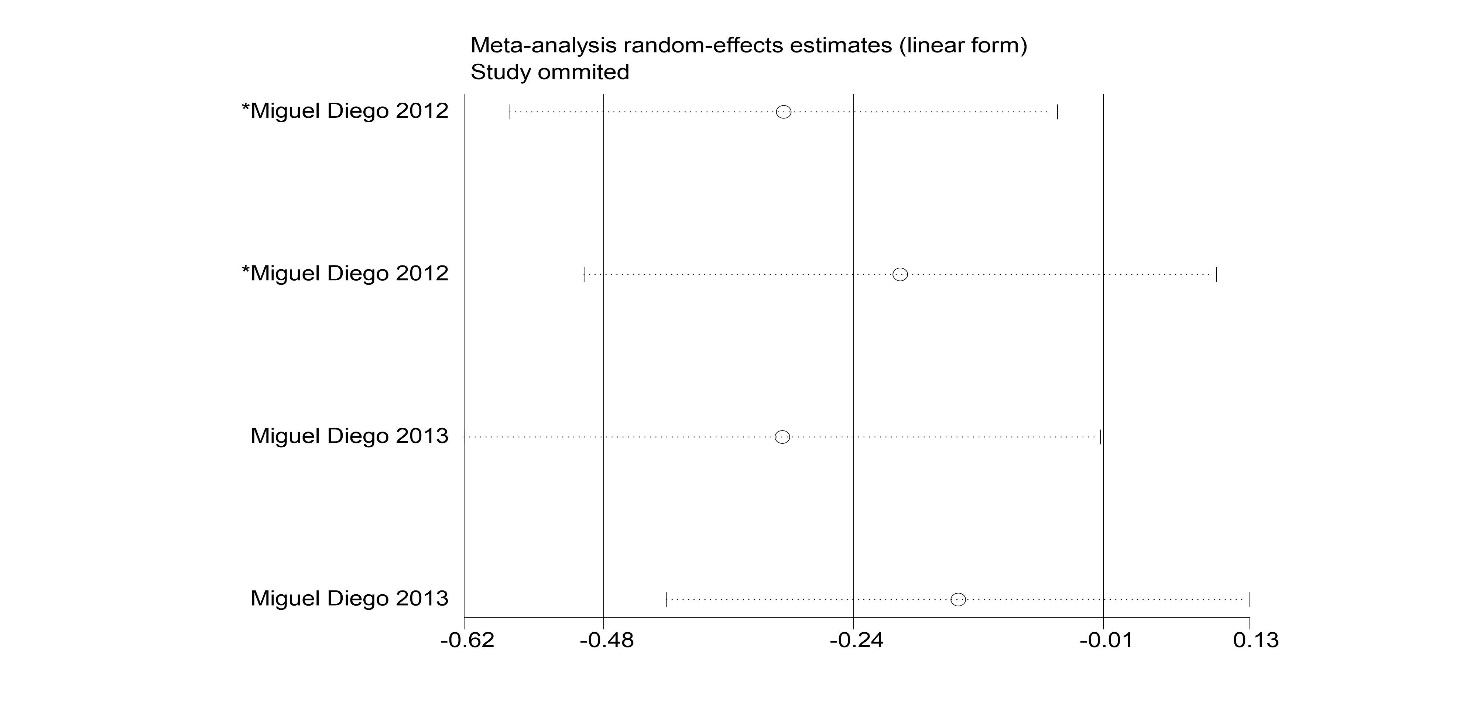
**

**(C)**

**
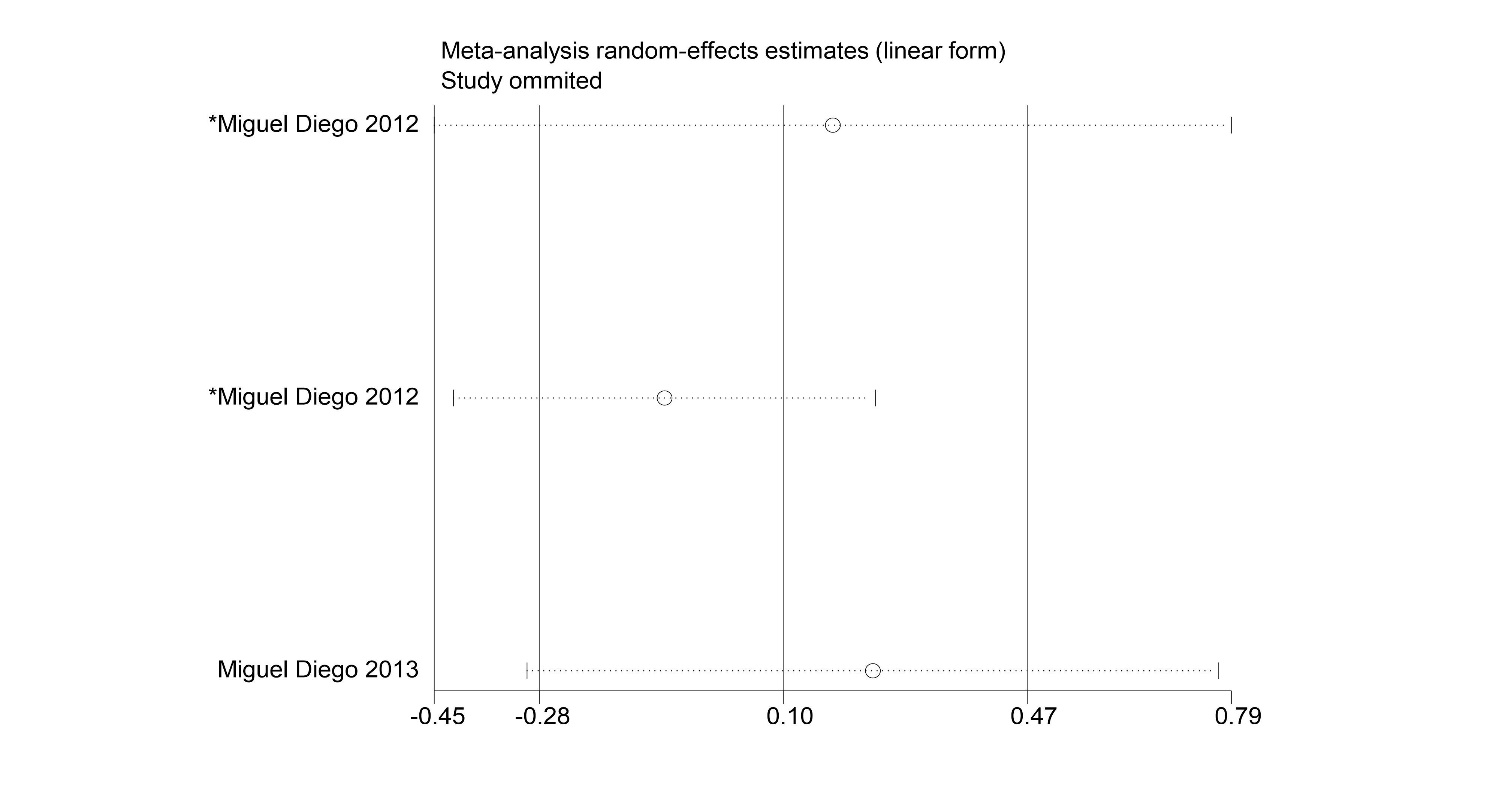
**

**(D)**

**
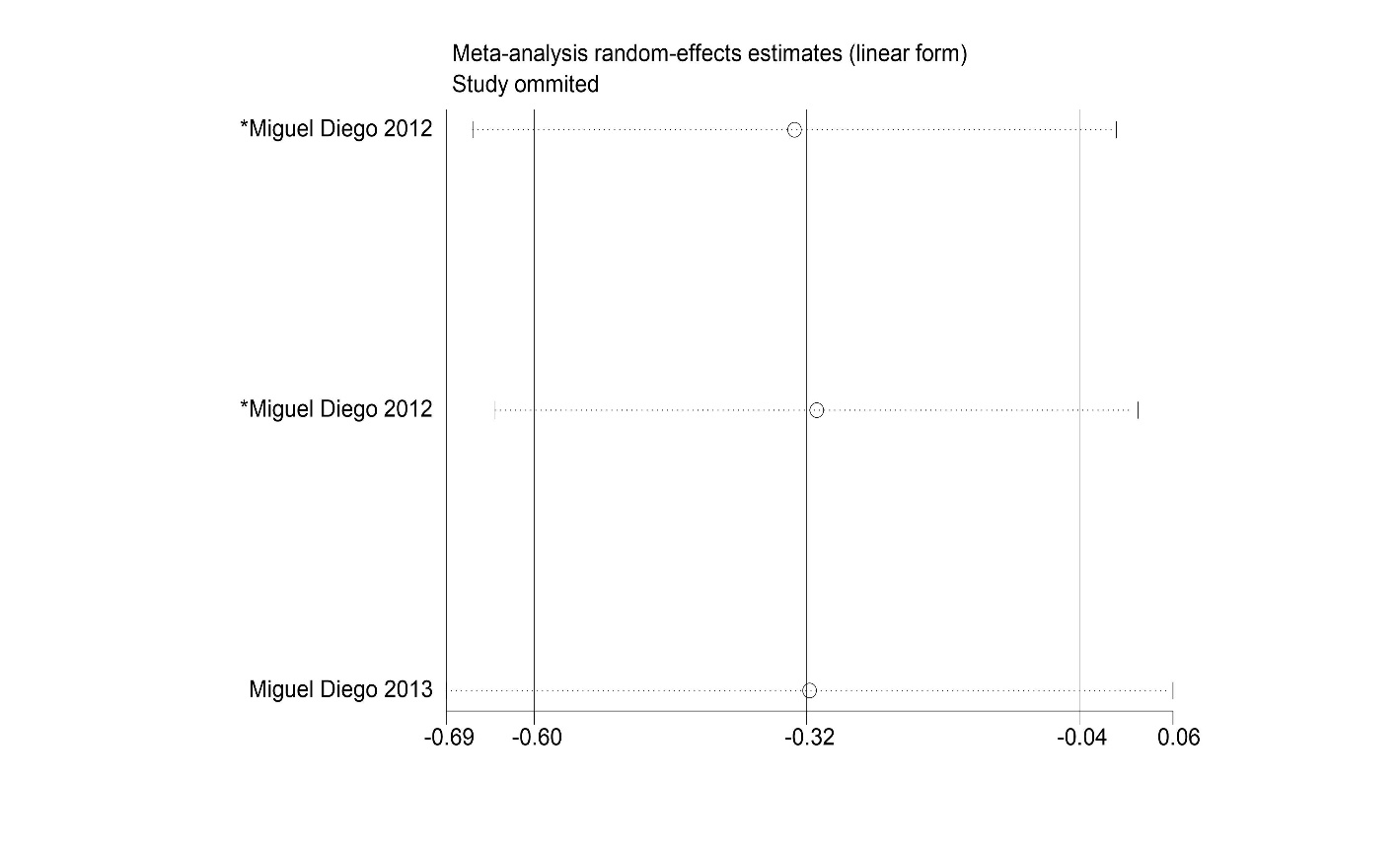
**

**(E)**

**Supplementary Figure 4.** **(A)** **Sensitivity analysis of HAMD; (B) Sensitivity analysis of BDI-II; (C) Sensitivity analysis of STAI; (D) Sensitivity analysis of Relationships (E) Sensitivity analysis of STAXI**

## Supplementary Tables

**Supplementary table 1. PRISMA Statement**

| **Section/topic** | **#** | **Checklist item** | **Reported on page #** |
| --- | --- | --- | --- |
| **TITLE** | | |  |
| Title | 1 | Identify the report as a systematic review, meta-analysis, or both. | Both |
| **ABSTRACT** | | |  |
| Structured summary | 2 | Provide a structured summary including, as applicable: background; objectives; data sources; study eligibility criteria, participants, and interventions; study appraisal and synthesis methods; results; limitations; conclusions and implications of key findings; systematic review registration number. | 2 |
| **INTRODUCTION** | | |  |
| Rationale | 3 | Describe the rationale for the review in the context of what is already known. | 2 |
| Objectives | 4 | Provide an explicit statement of questions being addressed with reference to participants, interventions, comparisons, outcomes, and study design (PICOS). | 2 |
| METHODS | | |  |
| Protocol and registration | 5 | Indicate if a review protocol exists, if and where it can be accessed (e.g., Web address), and, if available, provide registration information including registration number. | 2 |
| Eligibility criteria | 6 | Specify study characteristics (e.g., PICOS, length of follow-up) and report characteristics (e.g., years considered, language, publication status) used as criteria for eligibility, giving rationale. | 3 |
| Information sources | 7 | Describe all information sources (e.g., databases with dates of coverage, contact with study authors to identify additional studies) in the search and date last searched. | 3 |
| Search | 8 | Present full electronic search strategy for at least one database, including any limits used, such that it could be repeated. | 3 |
| Study selection | 9 | State the process for selecting studies (i.e., screening, eligibility, included in systematic review, and, if applicable, included in the meta-analysis). | 3 |
| Data collection process | 10 | Describe method of data extraction from reports (e.g., piloted forms, independently, in duplicate) and any processes for obtaining and confirming data from investigators. | 3 |
| Data items | 11 | List and define all variables for which data were sought (e.g., PICOS, funding sources) and any assumptions and simplifications made. | 3 |
| Risk of bias in individual studies | 12 | Describe methods used for assessing risk of bias of individual studies (including specification of whether this was done at the study or outcome level), and how this information is to be used in any data synthesis. | 3 |
| Summary measures | 13 | State the principal summary measures (e.g., risk ratio, difference in means). | 3 |
| Synthesis of results | 14 | Describe the methods of handling data and combining results of studies, if done, including measures of consistency (e.g., I2) for each meta-analysis. | 3 |
| Risk of bias across studies | 15 | Specify any assessment of risk of bias that may affect the cumulative evidence (e.g., publication bias, selective reporting within studies). | 3 |
| Additional analyses | 16 | Describe methods of additional analyses (e.g., sensitivity or subgroup analyses, meta-regression), if done, indicating which were pre-specified. | 3 |
| **RESULTS** | | |  |
| Study selection | 17 | Give numbers of studies screened, assessed for eligibility, and included in the review, with reasons for exclusions at each stage, ideally with a flow diagram. | 4 |
| Study characteristics | 18 | For each study, present characteristics for which data were extracted (e.g., study size, PICOS, follow-up period) and provide the citations. | 4 |
| Risk of bias within studies | 19 | Present data on risk of bias of each study and, if available, any outcome level assessment (see item 12). | 4 |
| Results of individual studies | 20 | For all outcomes considered (benefits or harms), present, for each study: (a) simple summary data for each intervention group (b) effect estimates and confidence intervals, ideally with a forest plot. | 4, 5 |
| Synthesis of results | 21 | Present results of each meta-analysis done, including confidence intervals and measures of consistency. | 4, 5 |
| Risk of bias across studies | 22 | Present results of any assessment of risk of bias across studies (see Item 15). | 4, 5 |
| Additional analysis | 23 | Give results of additional analyses, if done (e.g., sensitivity or subgroup analyses, meta-regression [see Item 16]). | 4, 5 |
| **DISCUSSION** | | |  |
| Summary of evidence | 24 | Summarize the main findings including the strength of evidence for each main outcome; consider their relevance to key groups (e.g., healthcare providers, users, and policy makers). | 5, 6 |
| Limitations | 25 | Discuss limitations at study and outcome level (e.g., risk of bias), and at review-level (e.g., incomplete retrieval of identified research, reporting bias). | 5 |
| Conclusions | 26 | Provide a general interpretation of the results in the context of other evidence, and implications for future research. | 7 |
| **FUNDING** | | |  |
| Funding | 27 | Describe sources of funding for the systematic review and other support (e.g., supply of data); role of funders for the systematic review. | 7 |

From: Moher D, Liberati A, Tetzlaff J, Altman DG, The PRISMA Group (2009). Preferred Reporting Items for Systematic Reviews and Meta-Analyses: The PRISMA Statement. PLoS Med 6(7): e1000097. doi:10.1371/journal. pmed1000097

For more information, visit: **www.prisma-statement.org**.

**Supplementary table 2. The search strategy of the PubMed database**

|  |  |
| --- | --- |
| #1 | Depressive disorders, major [mh] OR Major depressive disorders [mh] OR Major depressive disorder [mh] |
| #2 | Depressive* [tiab] OR MDD [tiab] |
| #3 | #1 OR #2 |
| #4 | Yoga [tiab] |
| #5 | Randomized controlled trial[pt] OR Controlled clinical trial[pt] |
| #6 | Randomized controlled trial[tiab] OR Controlled clinical trial[tiab] OR Randomized*[tiab] OR Randomly*[tiab] Random allocation[tiab] OR Trial[tiab] OR CCT [tiab] OR RCT [tiab] |
| #7 | #5 OR #6 |
| #8 | #3 AND #4 AND #7 |

Note: mh: MeSH; tiab: tittle/abstract; pt: publication type

**Supplementary Table 3. Risk of bias judgments for randomized controlled trials (RoB 2.0)**

| **ID** | **Bias arising from the randomization process** | **Bias due to deviations from intended intervention** | **Bias due to missing outcome data** | **Bias in measurement of the outcome** | **Bias in selection of the reported result** | **Overall** |
| --- | --- | --- | --- | --- | --- | --- |
| **Naga Venkatesha Murthy 2000** | Some concerns | Low | Low | Low | Low | Some concerns |
| **S. DAS 2005** | Some concerns | Low | Low | Low | Low | Some concerns |
| **Vedamurthachar 2006** | Some concerns | Low | Low | Low | Low | Some concerns |
| **Uddip Talukdar 2011** | Some concerns | Low | Low | Low | Low | Some concerns |
| **Miguel Diego 2012** | Some concerns | Low | Low | Low | Low | Some concerns |
| **Khushbu Rani 2012** | Low | Low | Low | Low | Low | Low |
| **Miguel Diego 2013** | Some concerns | Low | Low | Low | Low | Some concerns |
| **Miguel Diego 2013** | Some concerns | Low | Low | Low | Low | Some concerns |
| **Cheryl Bourguignon 2013** | Low | Low | Some concerns | Low | Low | Some concerns |
| **Patricia AnneKinser 2014** | Low | Low | Low | Low | Low | Low |
| **Caroline Nothdurfter 2014** | Some concerns | Low | Low | Low | Low | Some concerns |
| **Beth A. Lewis 2016** | Low | Low | Low | Low | Low | Low |
| **Renee Rivera 2017** | Low | Low | Low | Low | Low | Low |
| **Marna S. Barrett 2017** | Some concerns | Low | Low | Low | Low | Some concerns |
| **Chris C. Streeter 2017** | Low | Low | Low | Low | Low | Low |
| **Lisa A. Uebelacker 2017** | Low | Low | Low | Low | Low | Low |
| **Patricia L.Gerbarg 2018** | Low | Low | Low | Low | Low | Low |
| **Madhuri R. 2018** | Low | Low | Low | Low | Low | Low |
| **Madhuri R. 2018** | Low | Low | Low | Low | Low | Low |
| **Jolene Mui 2019** | Low | Low | Low | Low | Low | Low |
| **TAMMY M. 2019** | Low | Low | Low | Low | Low | Low |
| **Chris C. Streeter 2020** | Low | Low | Low | Low | Low | Low |
| **Praerna H. Bhargav2021** | Low | Low | Low | Low | Low | Low |
| **Esra Görgülü 2021** | Some concerns | Low | Low | Low | Low | Low |
| **Kankan Gulati 2021** | Low | Low | Low | Low | Low | Low |
| **Nicole R. Nugent 2021** | Low | Low | Low | Low | Low | Low |
| **Arun V. Ravindran2021** | Low | Low | Low | Low | Low | Low |
| **Ananya Srivastava 2021** | Some concerns | Low | Low | Low | Low | Some concerns |
| **Manjula Subbanna 2021** | Some concerns | Low | Some concerns | Low | Low | Some concerns |
| **Jessica L. West 2021** | Low | Low | Some concerns | Low | Low | Some concerns |
| **LIN Yihuan 2010** | Some concerns | Low | Low | Low | Low | Some concerns |
| **Wang Wenjuan 2020** | Low | Low | Low | Low | Low | Low |
| **Liang Jinmei 2017** | Some concerns | Low | Low | Low | Low | Some concerns |
| **LI Li 2019** | Low | Low | Low | Low | Low | Low |

**Supplementary Table 4. Eggers and Begg test**

| **Rating scale** | **Begg's Test** | **Egger's test** |
| --- | --- | --- |
| **HAMD** | 0.563 | 0.368 |
| **BDI-II** | 0.26 | 0.09 |
| **STAI** | 1 | 0.466 |
| **STAXI** | 0.296 | 0.488 |
| **Relationships** | 0.117 | 0.48 |

The results of egger test and Begg test mainly focus on the value of p, and it is generally believed that there is publication bias when p<0.05. In this case, the results of Begg test and Egger test were all >0.05, indicating no significant publication bias. In general, egger's test is slightly more efficient than egger's test.

**Supplementary Table 5. Meta-analysis of other outcome indicators**

| Indicator | Study | Heterogeneity | Cohen’ d | 95%CI |
| --- | --- | --- | --- | --- |
| BDI scores | vedamurthachar 2006  Arun V 2021 | I^2^=37.87% | -1.08 | (-1.58，-0.59) |
| Cortisol | Vedamurthachar 2006  Madhuri 2018 | I^2^=44.53% | -1.13 | (-1.66，-0.61) |
| IL-6 | Nicole R 2021  Madhuri R 2018 | I^2^=46.60% | -1.14 | (-1.63，-0.64) |
| MADRS scores | Esra Görgülü 2021  Arun V 2021 | I^2^=0.00% | -0.49 | (-0.83，-0.15) |
| STAXI | *Miguel Diego 2012  *Miguel Diego 2012  Miguel Diego 2013 | I^2^=0.00% | -0.32 | (-0.60，-0.04) |
